# Metal Coordination and Biological Screening of a Schiff Base Derived from 8-Hydroxyquinoline and Benzothiazole

**DOI:** 10.3390/pharmaceutics14122583

**Published:** 2022-11-24

**Authors:** Nádia Ribeiro, Pedro F. Farinha, Jacinta O. Pinho, Hugo Luiz, János P. Mészáros, Adelino M. Galvão, João Costa Pessoa, Éva A. Enyedy, Catarina Pinto Reis, Isabel Correia, Maria Manuela Gaspar

**Affiliations:** 1 Centro de Química Estrutural, Institute of Molecular Sciences and Departamento de Engenharia Química, Instituto Superior Técnico, Universidade de Lisboa, Av. Rovisco Pais, 1, 1049-001 Lisboa, Portugal; 2 Research Institute for Medicines, iMed.ULisboa, Faculty of Pharmacy, Universidade de Lisboa, 1649-003 Lisboa, Portugal; 3 MTA-SZTE Lendület Functional Metal Complexes Research Group, Department of Inorganic and Analytical Chemistry, University of Szeged, Dóm tér 7, H-6720 Szeged, Hungary

**Keywords:** metal complexes, hydrazone, proton dissociation constants, albumin binding, colorectal cancer, in vitro studies

## Abstract

Designing new metallodrugs for anticancer therapy is a driving force in the scientific community. Aiming to contribute to this field, we hereby report the development of a Schiff base (H_2_L) derived from the condensation of 2-carbaldehyde-8-hydroxyquinoline with 2-hydrazinobenzothiazole and its complexation with transition metal ions. All compounds were characterised by analytical and spectroscopic techniques, which disclosed their structure: [Cu(HL)Cl], [Cu(HL)_2_], [Ni(HL)(acetate)], [Ni(HL)_2_], [Ru(HL)Cl(DMSO)], [VO(HL)_2_] and [Fe(HL)_2_Cl(H_2_O)]. Different binding modes were proposed, showing the ligand’s coordination versatility. The ligand proton dissociation constants were determined, and the tested compounds showed high lipophilicity and light sensitivity. The stability of all complexes in aqueous media and their ability to bind to albumin were screened. Based on an antiproliferative in vitro screening, [Ni(HL)(acetate)] and [Ru(HL)Cl(DMSO)] were selected for further studies aiming to investigate their mechanisms of action and therapeutic potential towards colon cancer. The complexes displayed IC_50_ < 21 μM towards murine (CT-26) and human (HCT-116) colon cancer cell lines. Importantly, both complexes exhibited superior antiproliferative properties compared to the clinically approved 5-fluorouracil. [Ni(HL)(acetate)] induced cell cycle arrest in S phase in CT-26 cells. For [Ru(HL)Cl(DMSO)] this effect was observed in both colon cancer cell lines. Additionally, both compounds significantly inhibited cell migration particularly in the human colon cancer cell line, HCT-116. Overall, the therapeutic potential of both metal complexes was demonstrated.

## 1. Introduction

Cancer is one of the most challenging diseases and related deaths are estimated to rise as life expectancy increases [1]. Although relevant progresses in therapy have been attained over the past decades, the incidence and mortality continue to increase. Particularly, colorectal arises as the fourth most prevalent form of cancer both in the EU and the USA [2]. In Portugal, this malignancy has the highest incidence and represents the second most lethal type of cancer [3]. As such, knowledge about its pathophysiology and aetiology is crucial to design effective treatments. Colorectal cancer develops when the healthy colorectal epithelium suffers changes (due to genetic and environmental factors), which can lead to the formation of malignant adenomatous polyps that proliferate in an uncontrolled manner, increasing in size and invading nearby tissues. In the worst cases, they can reach lymphatic and blood vessels and spread to distant regions of the body (stages III and IV), increasing considerably the mortality [4]. In such conditions, chemotherapy plays a crucial role in tumour shrinkage and relief of symptoms, generally leading to better prognosis when compared to surgical resection alone [4]. The treatment of advanced stages of colorectal cancer includes a combination of multiple first-line drugs such as 5-fluorouracil (5-FU), capecitabine, leucovorin and oxaliplatin, a platinum-based metal complex [4,5]. Although effective, platinum metallodrugs exhibit several disadvantages, particularly systemic toxicity and tumour resistance to therapy [6,7]. In this regard, the search for other metal-based anticancer agents with improved safety profiles and different mechanisms of action has become an attractive area of study for colorectal management [7,8].

Schiff bases are quite attractive compounds due to their easy synthesis and structure versatility depending on the chosen module molecules. Moreover, they usually provide good chelating moieties for metal coordination. Both benzothiazole and 8-hydroxyquinoline are privileged scaffolds in medicinal chemistry [9,10], showing separately a wide range of biological activities such as antitumour [11,12,13,14,15,16], antitubercular, antimicrobial [17,18] and anti-neurodegenerative [19]. In addition, they have been also used as starting materials for the synthesis of various drugs. From our knowledge, a few examples have been described where the two features were combined [11]. The 2-position of benzothiazole has already been applied as a starting point to build Schiff bases with several aromatic aldehydes [11]. In contrast, the use of 2-carbaldehyde-8-hydroxyquinoline for this purpose has not yet been reported in the literature. 8-hydroxyquinoline-based benzothiazole derivatives have been mostly explored in the coordination to lanthanide ions, due to their luminescent properties [20], or as selective probes [21]. Coordination to copper(II) has also been exploited, with the prepared compounds exhibiting a rich chemistry, cytotoxic activity in the low micromolar range and artificial nuclease ability [22].

Recently, we reported a new Schiff base derived from the condensation of 2-carbaldehyde-8-hydroxyquinoline and 2-hydrazinobenzothiazole, which was characterised and used to bind zinc(II) [23]. Two metal complexes with 1:1 and 1:2 metal-to-ligand stoichiometry were obtained and the first one, [ZnL(acetate)], where L is the deprotonated form of the ligand, demonstrated higher antiproliferative properties and was selected for further studies. This Zn-complex was efficiently incorporated in long blood circulating and pH-sensitive liposomes preserving its cytotoxic activity towards murine and human colon cancer cells. The therapeutic potential of free and liposomal formulations of the Zn-complex was assessed in a syngeneic murine colon cancer model. While the complex in the free form did not exert antitumour effect, an impaired tumour progression was achieved for the compound loaded in liposomes. Importantly, the nanoformulation displayed similar efficacy as the positive control 5-FU, using a three-fold lower therapeutic dose [23]. These results inspired us to further study the application of this Schiff base and its metal complexes in anticancer research.

Thus, the main objectives of this work are the design of new metallodrugs using this Schiff base. We present the characterisation of the Schiff base; the study of its lipophilicity and pH-dependent solution speciation; its complexation towards several relevant metal ions, namely copper(II), nickel(II), iron(III), ruthenium(III) and vanadium(IV); the screening of their cytotoxic activity; and the evaluation of their potential as anticancer drugs elucidating underlying mechanisms of action. We believe that this work provides an in-depth understanding of the coordination chemistry of Schiff base hydrazones towards different metal ions with different oxidation states and physicochemical properties. Moreover, their potential as anticancer agents is highlighted.

## 2. Materials and Methods

### 2.1. Materials and Apparatus 

2-carbaldehyde-8-hydroxyquinoline, 2-hydrazinobenzothiazole and Cu(II) chloride, Ni(II) acetate, V(IV)O acetylacetonate (acac) and Fe(III) chloride were obtained from Sigma and used without further purification. The precursor, hydrogen *trans*-bis(dimethyl sulfoxide) tetrachlororuthenate(III), [(DMSO)_2_H][Ru(DMSO)_2_Cl_4_] was synthetised according to a literature procedure [24]. All solvents were of *p.a.* grade and used as received. Phosphate buffered saline (PBS) was obtained from Sigma as readily soluble tablets and HEPES (4-(2-hydroxyethyl)-1-piperazineethanesulfonic acid) was purchased from Sigma. The water used in all studies with biomolecules was doubly deionised in a MilliQ system. The spectroscopic data were visualised and treated using Spectragryph software v. 1.2.15 [25]. Elemental analysis for C, H, N and S were carried on a FISONS EA 1108 CHNS-O apparatus at *Laboratório de Análises of Instituto Superior Técnico*. A 500-MS Varian Ion Trap Mass Spectrometer was used to measure ESI-MS spectra of methanolic solutions of the compounds in both positive and negative ion modes. A Bruker Avance II + 400 (UltraShieldTM Magnet) spectrometer operating at 400.13 MHz for proton and at 81 MHz for carbon was used to obtain the ^1^H and ^13^C NMR spectra. The chemical shifts are reported in ppm using tetramethylsilane as internal reference. A Perkin Elmer Lambda 35 spectrophotometer was used to measure the UV-vis electronic absorption spectra, while in the infrared region a JASCO FT/IR 4100 spectrophotometer was used. The first derivative X-band EPR spectra of the frozen solutions at ca. 100 K were recorded on a Bruker ESP 300E operated at ≈ 9.51 GHz with a frequency modulation of 100 KHz.

### 2.2. Synthesis

#### 2.2.1. Synthesis of (E)-2-((2-(benzo[d]thiazol-2-yl)Hydrazono)Methyl)Quinolin-8-ol (H_2_L)

The synthesis was reported on a previous publication [23] and only its characterisation will be described. Yield: 75–90%. Elemental analyses C, 61.6%; H, 3.8%; N, 16.8%; S, 9.6% (calc. for C_17_H_12_N_4_OS∙0.5 H_2_O: C, 61.99%; H, 3.98%; N, 17.01%; S, 9.73%). ESI-MS *m/z* 321.20 (cald: 321.08) [C_17_H_12_N_4_OS+H]^+^, *m/z* 319.47 (cald: 319.07) [C_17_H_12_N_4_OS−H]^−^. ^1^H NMR [400 MHz, DMSO_*d_6_*, δ (ppm)]: 12.75 ppm (NH); 9.82 ppm (OH); 8.34 ppm (HC=N); hydroxyquinoline moiety 7.13, 7.44, 7.41, 8.35, 8.04; benzothiazole moiety: 7.83, 7.16, 7.34, 7.5. ^13^C NMR [100.6 MHz, DMSO_*d_6_*, δ (ppm)]: 144.10 ppm (imine); hydroxyquinoline moiety: 138.13, 153.57, 112.16, 128.08, 117.80, 128.58, 136.6, 117.25, 151.42; benzothiazole moiety: 161.86, 130.19, 121.65, 122.08, 126.11, 120, 151.4. Soluble in dimethylformamide (DMF) and dimethyl sulfoxide (DMSO).

#### 2.2.2. Synthesis of the Copper(II) Complexes 

In a round bottom flask, 0.25 mmol of H_2_L was suspended in methanol (MeOH), deprotonated with 0.25 mmol of CH_3_ONa and the yellow mixture was stirred at room temperature (r. t.) for ca. 20 min. In a small vial, 1 eq. (1:1) or 0.5 eq. (2:1) of CuCl_2_·2H_2_O was dissolved in MeOH and added to the previous mixture, which immediately became red. The reaction mixture was left under stirring at r. t. overnight. The resulting solids were collected by filtration, washed with ice-cold MeOH and dried under vacuum in a desiccator over silica-gel.

[Cu(HL)Cl]—Reddish-brown solid. Yield: 87.5 mg (85%). Elemental analyses C, 45.6%; H, 2.5%; N, 12.2%; S, 7.8% (cald. for C_17_H_11_ClCuN_4_OS∙0.5 NaCl: C, 45.62%; H, 2.48%; N, 12.52%; S, 7.16%). ESI-MS *m/z* 382.01 (cald: 381.99) [Cu(HL)]^+^, *m/z* 459.47 (cald: 458.99) [[Cu(HL)(Cl)]+ACN+H]^+^. Soluble in DMF and DMSO.

[Cu(HL)_2_]—Reddish-brown solid. Yield: 123.6 mg (88%). Elemental analyses C, 55.8%; H, 3.1%; N, 15.0%; S, 8.7% (cald. for C_32_H_22_CuN_8_O_2_S_2_∙0.5 NaCl: C, 55.83%; H, 3.03%; N, 15.32%; S, 8.77%). ESI-MS *m/z* 699.76 (cald: 700.05) [Cu(HL)_2_−H]^−^, *m/z* 701.45 (cald: 702.07) [Cu(HL)_2_+H]^+^, *m/z* 764.65 (cald: 765.08) [[Cu(HL)_2_]+ACN+Na]^+^. Soluble in DMF and DMSO.

#### 2.2.3. Synthesis of the Nickel(II) Complexes

In a two-entry reaction vessel 0.25 mmol of H_2_L was suspended in ethanol (EtOH) and deprotonated with KOH in MeOH. In a vial, 1 eq. (1:1) or 0.5 eq. (2:1) of Ni(CH_3_COO)_2_·3H_2_O was dissolved in EtOH and added dropwise to the ligand solution and allowed to reflux for 5 h. The reaction mixture was left overnight in the freezer. The resulting solids were collected by filtration, washed with ice-cold EtOH and dried under vacuum in a desiccator over silica-gel.

[Ni(HL)(acetate)]—Red solid. Yield: 74.8 mg (68.5%). Elemental analyses C, 49.4%; H, 3.3%; N, 12.2%; S, 6.8% (cald. for C_19_H_14_N_4_O_3_SNi∙1.25 H_2_O: C, 49.65%; H, 3.62%; N, 12.19%; S, 6.98%). ESI-MS *m/z* 454.92 (cald: 455.13) [[Ni(HL)(acetate)]+NH_4_]^+^, *m/z* 377.21 (cald: 377.00) [Ni(HL)]^+^. Soluble in DMF and DMSO.

[Ni(HL)_2_]—Orange solid. Yield: 125 mg (89.6%). Elemental analyses C, 56.7%; H, 3.2%; N, 15.0%; S, 8.8% (cald. for C_34_H_22_N_8_O_2_S_2_Ni∙1H_2_O∙0.25 EtOH: C, 57.00%; H, 3.54%; N, 15.41%; S, 8.82%). ESI-MS *m/z* 696.6 (cald: 697.07) [Ni(HL)_2_+H]^+^, *m/z* 694.7 (cald: 695.06) [Ni(HL)_2_−H]^−^. ^1^H NMR [400 MHz, DMSO_*d_6_*, δ (ppm)]: 8.34 (2H, HC=N); 9.79 (2H, OH); 8.35 (2H); 8.04 (2H); 7.83 (2H); 7.53 (2H); 7.44–7.34 (6H); 7.16 (2H); 7.13 (2H). Soluble in DMF and DMSO.

#### 2.2.4. Synthesis of the Ruthenium(II) Complex

The procedure was similar to the one used for the Ni-complexes, but 1 eq. of triethylamine (Et_3_N) was used to deprotonate the ligand and the metal precursor used was [(DMSO)_2_H][*trans*-Ru(DMSO)_2_Cl_4_] [24].

[Ru(HL)Cl(DMSO)]—Dark violet solid. Yield: 88.4 mg (66.3%). Elemental analyses C, 41.6%; H, 3.3%; N, 9.9%; S, 11.8% (cald. for C_19_H_17_ClRuN_4_O_2_S_2_∙H_2_O: C, 41.34%; H, 3.47%; N, 10.15%; S, 12.01%). ESI-MS *m/z* 646.6 (cald: 646.94) [[Ru(HL)Cl(DMSO)]+TFA−H]^−^. ^1^H NMR [400 MHz, DMSO_*d*_6_, δ (ppm)]: broad signal at 10.1 ppm (OH); 8.38 ppm (HC=N); hydroxyquinoline moiety 7.15, 7.43, 7.46, 8.07, 8.40; benzothiazole moiety: 7.18, 7.36, 7.51, 7.84. ^13^C NMR [100.6 MHz, DMSO_*d*_6_, δ (ppm)]: 143.7 ppm (imine); hydroxyquinoline moiety: 112.5, 117.3, 117.7, 128, 137; benzothiazole moiety: 121.5, 122, 126, 128, 130. Soluble in DMF and DMSO.

#### 2.2.5. Synthesis of the Oxidovanadium(IV) Complex

The procedure was similar to the one used for the Ni(II) complexes, although VO(acac)_2_ was used as the metal precursor. The solutions were prepared with degassed solvents and the reaction was performed under nitrogen atmosphere.

[VO(HL)_2_]—Yellow-greenish solid. Yield: 108 mg (77%). Elemental analyses C, 57.2%; H, 3.2%; N, 15.3%; S, 9.2% (cald. for C_34_H_22_N_8_O_3_S_2_V·0.25H_2_O·0.25 EtOH: C, 57.42%, H, 3.35%, N, 15.53%, S, 8.88%). ESI-MS *m/z* 703.7 (cald: 704.06) [[VO(HL)_2_]−H]^−^, *m/z* 705.7 (cald: 706.08) [[VO(HL)_2_]+H]^+^. Soluble in DMF and DMSO, and slightly soluble in methanol.

#### 2.2.6. Synthesis of the Iron(III) Complex

The procedure was similar to the one used for the Ni(II)-complexes, but 2 eq. of Et_3_N were used to deprotonate the ligand and the metal precursor used was FeCl_3_·6H_2_O.

[Fe(HL)_2_Cl(H_2_O)]—Dark purple solid. Yield: 128 mg (81.5%). Elemental analyses C, 54.5%; H, 3.0%; N, 14.7%; S, 8.6% (cald. for C_34_H_24_ClFeN_8_O_3_S_2_: C, 54.59%; H, 3.23%; N, 14.98%; S, 8.57 ESI-MS *m/z* 693.9 (cald: 694.07) [Fe(HL)_2_]^+^, *m/z* 692.1 (cald: 692.05) [[Fe(HL)_2_]−2H]^−^. Soluble in DMF and DMSO.

### 2.3. UV-Visible Spectrophotometry

The pH-potentiometric titrations for the calibration of the electrode system were performed in 30% (*v*/*v*) and 60% (*v*/*v*) DMSO/H_2_O solvent mixtures using 0.10 M KCl background electrolyte at 25.0 ± 0.1 °C as described in our previous works [26,27]. 

A Thermo Evolution 220 spectrophotometer was used to record the UV-visible (UV-vis) spectra in the 260 to 750 nm window. The path length was varied between 2 and 50 mm. As a result of the limited solubility of the compounds in water, the spectrophotometric titrations were performed to determine the proton dissociation constants (p*K*_a_) of the ligand and its complex at 5 μM concentration using 5 cm path length. Due to the light sensitivity of the studied compounds, the samples were covered and were kept in the dark during the titrations. The starting sample volume was 50 mL. These titrations were performed in the pH range 2.0–12.5 (30% (*v*/*v*) DMSO/H_2_O) and 2.0–14.0 (60% (*v*/*v*) DMSO/H_2_O). Samples were deoxygenated by bubbling purified argon through them for approximately 10 min prior to the measurements and argon was also passed over the solutions during the spectrophotometric titrations. Calculation of the proton dissociation constants was performed with the computer program PSEQUAD [28].

For the light sensitivity studies, DMSO solutions were prepared. The starting sample was separated equally for more portions, one was irradiated with diffuse light, while other portions were stored in the dark and measured only once.

### 2.4. Lipophilicity Measurements

The distribution coefficient values (*D*_7.4_) of the compounds were determined by the traditional shake-flask method in *n*-octanol/buffered aqueous solution at pH 7.40 (10 mM HEPES buffer, in the absence or in the presence of 0.10 M KCl) at 25.0 ± 0.2 °C. The samples and stock solutions were protected from light until the spectrophotometric concentration determination. The ligand and complex were dissolved at 150 and 100 μM concentrations in *n*-octanol, respectively. *n*-Octanol was presaturated with the HEPES solution with the given KCl concentration. The aqueous solution and *n*-octanol ratio was 9:1 (instead of the usual 1:1 ratio due to the high lipophilicity of the compounds). The samples were mixed with vertical rotation (~20 rpm) for 4 h, then were centrifuged at 2415 *g* for 8 min. After separation, UV-vis spectra of the compounds in the *n*-octanol phase were recorded and compared to the spectra of the *n*-octanol stock solution. *D*_7.4_ values were calculated as in the following Equation (1):

(1) $$D_{7.4}=\frac{A_{oct,1}}{A_{oct,0}-A_{oct,1}}\times \frac{V_{water\;phase}}{V_{octanol\;phase}}$$

where *A_oct_*_,0_ and *A_oct_*_,1_ are the absorbance values of the *n*-octanol phase before and after separation, respectively, *V_water phase_* and *V_octanol phase_* are the volumes of the two phases.

### 2.5. ^1^H NMR Spectroscopy

^1^H NMR spectroscopic studies for the ligands and the ruthenium complex were carried out on a Bruker Avance III HD Ascend 500 Plus instrument. DMSO_*d*_6_ and 60% (*v*/*v*) DMSO_*d*_6_/HEPES mixture were used as solvent. DMSO_*d*_6_ was used as reference (*δ* = 2.50 ppm) and WATERGATE method was applied to suppress the solvent resonance in the aqueous samples. The samples contained the ligand or the complex in 200 μM–2 mM concentration.

### 2.6. Stability Assays under Aqueous Conditions

The complexes were monitored for their stability in PBS, pH 7.4 at 25 °C in the absence and presence of equimolar amounts of bovine serum albumin (BSA). Stock solutions of each complex were prepared in DMSO and then diluted in PBS in order to obtain final concentrations of 20 to 30 µM and ensuring that the organic solvent was less than 1% (*v*/*v*). For the assays with BSA, stock solutions of the protein were prepared in PBS using 20 mg of lyophilised powder in 5 mL solvent, which were allowed to hydrate for 24 h in the refrigerator. Samples were monitored by UV-vis absorption spectroscopy for 6 h and a final measurement was also taken after 24 h.

### 2.7. Fluorescence Assays 

Based on 3D scans, it was possible to conclude that the ligand, the Ru-complex and their HSA adducts are not fluorescent in 5% (*v*/*v*) DMSO/HEPES mixture. Prior to all spectrofluorometric measurements, samples were protected from light.

The concentration of BSA in the experiments was ca. 1.5 μM (in aqueous PBS). The fluorescence emission spectra were recorded between 310 and 575 nm, with λ_ex_ = 295 nm. Successive additions of the complexes were done directly to the cuvette (optical path 1 cm) and the fluorescence emission spectra were recorded for each one under the same conditions. Blank assays for each sample, consisting in solutions with the same concentration of complex and no BSA, were recorded and subtracted to the corresponding emission spectra containing the fluorophore. The UV-vis spectra of the sample solutions were used to correct the emission spectra, minimizing inner filter and reabsorption phenomena interference [29,30,31].

### 2.8. Circular Dichroism Spectroscopy with Bovine Serum Albumin

The concentration of BSA in the experiments was ca. 20–25 μM (in aqueous PBS). The circular dichroism (CD) spectra are an average of three scan accumulations, recorded between 300 and 500 nm, with bandwidth of 1 nm and response of 2 s, using a scanning speed of 100 nm/min. Successive additions of the complexes stock solutions in DMSO were done directly to the cuvette (1 cm) and the CD spectra were recorded under the same conditions after 10 min equilibration. Induced CD spectra were obtained as the CD spectrum of compound-BSA mixture minus the CD spectrum of BSA alone and are expressed as ellipticity in millidegrees. 

### 2.9. Cell Lines Culture Conditions 

Murine colon cancer CT-26 (ATCC^®^ CRL-263™) cells were cultured in RPMI-1640, supplemented with 10% foetal bovine serum (FBS) and 100 IU/mL of penicillin and 100 μg/mL streptomycin (Gibco, Thermo Fisher Scientific, Waltham, MA, USA), hereafter designated as complete culture medium. Human colon cancer HCT-116 (ATCC^®^ CCL-247™), human melanoma A375 (ATCC^®^ CCL-1619™), murine melanoma B16F10 (ATCC^®^ CCL-6475™) cells and human epidermal keratinocytes (HaCaT) were maintained in Dulbecco’s modified Eagle’s medium (DMEM) with high-glucose (4500 mg/L) (Sigma-Aldrich, St. Louis, MO, USA), supplemented with 10% FBS and 100 IU/mL of penicillin and 100 μg/mL streptomycin (Gibco, Thermo Fisher Scientific, Waltham, MA, USA), also designated as complete culture medium. The cell lines were maintained at 37 °C in a 5% CO_2_ environment and cells were trypsinised every 2–3 days when 80% confluence was reached.

### 2.10. In Vitro Antiproliferative Activity and Viability

The MTT [32] and Guava ViaCount assays [33,34] were used to assess cell viability in the absence (negative control) or presence of increasing concentrations of [Ni(HL)(acetate)], [Ru(HL)Cl(DMSO)] and 5-FU (positive control). Briefly, cells were plated at a concentration of 5 × 10^4^ cells/mL in 96-well plates (200 μL/well) and cultured for 24 h in the culture conditions described above. Afterwards, supernatant was removed, and cells were incubated with [Ni(HL)(acetate)] and [Ru(HL)Cl(DMSO)] at concentrations ranging from 0.5 to 40 μM or from 0.5 to 100 μM for the 5-FU (positive control). Negative controls were cells maintained in complete culture medium. After 48 h, culture medium was discarded, cells were washed twice with PBS and 50 μL of MTT at 0.5 mg/mL in incomplete medium was added to all wells. After a 2 to 3 h incubation period at 37 °C and 5% CO_2_ environment, 100 μL DMSO was added to each well to solubilise the formazan crystals, and absorbance was measured at 570 nm using a microplate reader Model 680 (Bio-Rad, CA, USA). Between three to four independent experiments were carried out, with six replicates *per* condition. GraphPad Prism^®^8 (GraphPad Software, San Diego, CA, USA) was used to analyse cell proliferation and values were plotted and fit to a standard log dose-response curve. The IC_50_ values were calculated using Equation (2) [35]:

log IC_50_ = log X_1_ + {[(Y_1_ − (Y_0_)/2)]/(Y_1_ − Y_2_)} × (log X_2_ − log X_1_) (2)

where, Y_0_/2 is the half-cell density of the negative control; Y_1_ is the cell density above Y_0_/2; X_1_ is the concentration corresponding to Y_1_; Y_2_ is the cell density below Y_0_/2; X_2_ is the concentration corresponding to Y_2_. The IC_50_ was determined by linear interpolation between X_1_ and X_2._

For Guava ViaCount assay, cells were plated at a concentration of 1 × 10^5^ cells/mL in 24-well plates (1 mL/well) and cultured for 24 h in the culture conditions described above [33]. CT-26 cells were incubated with [Ni(HL)(acetate)] or [Ru(HL)Cl(DMSO)] at concentrations of 5 and 10 μM or 10 and 20 μM, respectively. For the HCT-116 cell line, the tested concentrations were 5 and 7.5 μM, and 7.5 and 12.5 μM, respectively. The positive control group, 5-FU, was tested at 0.5 and 2 μM, and at 20 and 40 μM for CT-26 and HCT-116, respectively. Negative control group was constituted by cells in the presence of complete culture medium. After a 48 h incubation period, cell culture supernatants were collected, and adherent cells were detached using TrypLE™ Express (Invitrogen). Detached cells were then added to supernatants and the wells were washed using PBS with 2% FBS to recover any remaining cells. The recovered suspension was added to the previously collected cells and supernatants and centrifuged for 5 min at 650× *g*. The supernatants were discarded, and cells were suspended in PBS with 2% FBS. Then, on a 96-well plate, 20 μL of cell suspension was incubated with 180 μL of Guava ViaCount^®^ reagent (Merck Millipore, Darmstadt, Germany) for 5 min at r. t. Using a Guava EasyCyte 5HT Flow cytometer (Guava Technologies, Inc., Hayward, CA, USA), data were collected and analysed with the ViaCount software module with the acquisition of 10,000 events *per* sample. GraphPad Prism^®^8 was used to analyse cell proliferation (GraphPad Software, San Diego, CA, USA). Two independent experiments were carried out, with three replicates *per* condition.

### 2.11. Cell Cycle Analysis

To understand the potential effects of [Ni(HL)(acetate)] and [Ru(HL)Cl(DMSO)] on cell cycle progression, CT-26 and HCT-116 cells were used [36]. Briefly, cells were plated at a density of 5 × 10^4^ cells/mL in 6-well plates (2 mL) and allowed to grow for 24 h at 37 °C with 5% CO_2_. CT-26 cells were then incubated with [Ni(HL)(acetate)] or [Ru(HL)Cl(DMSO)] at concentrations of 5 and 10 μM or 10 and 20 μM, respectively. For HCT-116 cell line, the tested concentrations were 5 and 7.5 μM, and 7.5 and 12.5 μM for [Ni(HL)(acetate)] and [Ru(HL)Cl(DMSO)], respectively. The positive control group, 5-FU, was tested at 0.5 and 2 μM and 20 and 40 μM for CT-26 and HCT-116, respectively. Negative control group was constituted by cells in the presence of complete culture medium. After a 24 h incubation period, cells were collected by centrifugation at 850× *g* for 5 min at 4 °C, suspended in 500 µL of ice-cold PBS, and fixed with 500 µL ice-cold 80% ethanol (−20 °C), added drop-by-drop, under gentle mixing. Prior to the analysis, samples were kept at 4 °C for at least 16 h. On the day of data collection, cells were centrifuged at 850× *g* and suspended in PBS with 25 µg/mL PI (Fluka, Sigma-Aldrich) and 50 µg/mL RNase A (Sigma-Aldrich), then incubated at 37 °C for 30 min. The Guava easyCyte™ Flow Cytometer (Merck Millipore, Darmstadt, Germany) was used to collect the data corresponding to 10,000 events *per* sample. The ModFit LT™ 5.0 software was used to analyse the data and the percentages of cells in each cell cycle phase was determined (Verity Software House, Topsham, ME, USA).

### 2.12. Migration Assay

To assess cellular migration in the presence or absence of tested compounds, HCT-116 and CT-26 cells were plated at 1.5 × 10^5^ and 3 × 10^5^ cells/mL in a 12-well plate (1 mL/well) and were allowed to adhere and grow until reaching around 80–90% confluence [36,37]. A sterile 10 μL pipette tip was used to wound the cell monolayer, followed by a washing step with PBS to remove debris. Then, cells were incubated with the tested compounds suspended in RPMI-1640 (CT-26) or DMEM media (HCT-116) supplemented with 2% FBS and 100 IU/mL of penicillin and 100 μg/mL streptomycin (Gibco, Thermo Fisher Scientific, Waltham, MA, USA), and hereafter designated as RPMI 2% and DMEM 2%, respectively. Under the above mentioned culture conditions, CT-26 cells were incubated with [Ni(HL)(acetate)] or [Ru(HL)Cl(DMSO)] at concentrations of 10 and 15 μM, respectively. For the HCT-116 cells, the tested concentrations were 12.5 and 15 μM for [Ni(HL)(acetate)] and [Ru(HL)Cl(DMSO)], respectively. The positive control, 5-FU, was tested at 1.5 and 40 μM for CT-26 and HCT-116, respectively. Negative control group was constituted by cells maintained in RPMI 2% (CT-26) and DMEM 2% (HCT-116). Images of the wound closure were taken beneath the stage of a Zeiss Axiovert 200 inverted microscope, using a 10× objective and a digital camera (CoolSNAP EZ, Photometrics, Huntington Beach, CA, USA) and recorded by the Metafluor Software at 0, 2, 4, 8, 18, 20 and 22 h following cell monolayer scratching (Molecular Devices, Sunnyvale, CA, USA). ImageJ (https://imagej.net (accessed on 15 September 2022)) was used to analyse the images, and wound closure was normalised to the original wound area at time 0 h using Equation (2), where *W*_0_ represents the wound width at time 0 h and *W_t_* represents the wound width at the studied timepoints. Three independent experiments were performed, with three replicates for each tested condition.

(3) $$Wound\;closure\;\left(\%\right)=\frac{W_{0}-W_{t}}{W_{0}}\times 100$$

### 2.13. Statistical Analysis

Results are expressed as mean ± standard deviation (SD). Statistical analysis was performed with two-way ANOVA followed by Dunnett post-hoc test using GraphPad Prism^®^8 for Windows (GraphPad Software, San Diego, CA, USA). *p* < 0.05 was considered statistically significant.

### 2.14. Computational Methodologies

All theoretical calculations were of the density functional theory/ time-dependent density functional theory (DFT/TDDFT) type, carried out using GAMESS-US version R3 [38]. A range corrected LC-BPBE (μ = 0.20) functional as implemented in GAMESS-US [38] was used in both ground- and excited-state calculations. The solvent was included using the polarisable continuum model with the solvation model density to add corrections for cavitation, dispersion, and solvent structure. In TDDFT calculation of FC (Franck-Condon) excitations the dielectric constant of the solvent was split into a “bulk” component and a fast component, which is essentially the square of the refractive index. In “adiabatic” conditions only the static dielectric constant is used. A SBKJC effective core potential and corresponding-31G basis set was used in either DFT or TDDFT calculations.

## 3. Results and Discussion

### 3.1. The Ligand Precursor

**Figure 1:**
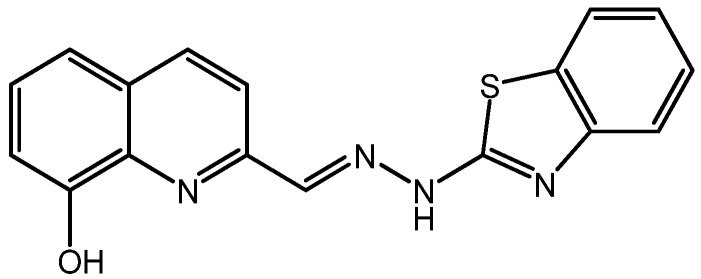
Molecular structure of H_2_L, a Schiff base derived from 8-hydroxyquinoline and benzothiazole.

The condensation reaction of 2-carbaldehyde-8-hydroxyquinoline with 2-hydrazinobenzothiazole provided the ligand precursor, H_2_L, as shown in Figure 1. Since its characterisation has been already published by us [23], details are included in Supplementary Material and here we report only the new solution studies. 

Spectroscopic and elemental studies confirmed the structure and purity of the prepared Schiff base. In the electronic absorption spectrum measured in DMSO (Figure S1a), it is possible to identify three maxima at λ = 265 nm (ε = 2.60 × 10^4^ M^−1^cm^−1^), 368 nm (ε = 2.87 × 10^4^ M^−1^cm^−1^) and 476 nm (ε = 9.78 × 10^3^ M^−1^cm^−1^). According to the definition presented by Kasha and Rawls for the electronic transitions [39], the two bands in the UV correspond to π→π* transitions within the quinoline and benzothiazole chromophores. The band at 476 nm is more difficult to assign, and a study carried in the absence of light, and described below, indicates that H_2_L is light sensitive. 

Nevertheless, UV-vis spectroscopic studies revealed an interesting behaviour for H_2_L. The lower energy band is only present in solvents of higher donor number (DMSO and DMF). Its extinction coefficient decreases as the concentration increases, while the band at 372 nm increases, this being more pronounced in DMSO. Concentration dependence effects have been described for donor–acceptor intramolecular interactions [40,41]. Considering the Gutmann basicity scale [42], only DMSO and DMF possess donor numbers (DN) which seem to be sufficiently high to deprotonate the hydrazone moiety of HL. These experimental results are well described by DFT calculations, which showed that only the deprotonated species possesses the band in the visible range (notice that the calculations indicate that this deprotonation refers to the NH of the hydrazone moiety and not the hydroxyl group in the 8-hydroxyquinoline). 

**Figure 2:**
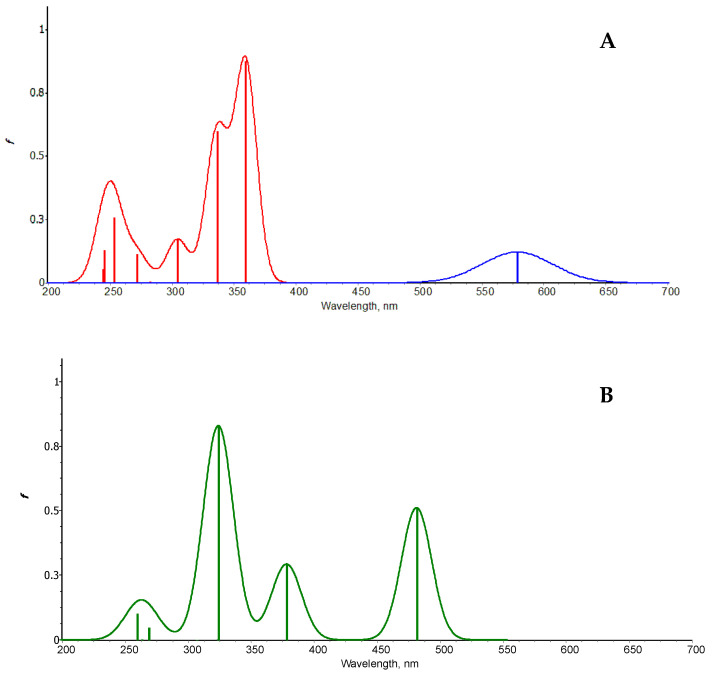
TDDFT simulated spectra of (**A**) H_2_L in DMSO. Red—absorption, blue—emission (after ESPT) and (**B**) HL^−^ in DMSO. f is the oscillator strength.

DFT calculations were used to access the structural conformations of H_2_L in both gas phase and DMSO. Figure S2 shows the tested conformers, isomer D, presented in Figure 1, being the most stable by about 9 kJ/mol (DMSO), which corresponds to being almost (97.5% Boltzmann distribution) the only conformer present in solution. The DMSO TDDFT simulated spectrum, Figure 2A, is identical to the experimental one with absorption bands at 250 and 360 nm plus a shoulder at 340 nm and a weak band at 305 nm. Probing the S1 potential energy surface (PES) showed an internal excited state proton transfer (ESPT) of the hydroxyl proton to the neighbour nitrogen creating a zwitterionic structure, which has an emission at 580 nm close to the experimentally observed. However, the absorption band at 476 nm is absent in simulations, which prompted us to find other possible species in solution. The deprotonation of H_2_L leads to an anionic base with an extra absorption at 480 nm, Figure 2B, also observed in the experimental spectrum. The combined simulated data suggest that in high donor number, high dielectric constant solvents, the separation of charges of the zwitterionic form are stabilised leading to continuous irradiation to the formation of this species, without a band at 480 nm. Putting the sample in darkness re-establishes the pH equilibrium and the band of anionic base resulting from proton transfer to the solvent. Simulations also predict a 15 nm red shift of the 360 nm band upon formation of the zwitterionic form, in good agreement with the shift observed under continuous irradiation. Since emission is from the zwitterionic form, H_2_L only has emission in solvents, such as DMSO, that can stabilise the charges in this species.

Fluorescence characterisation showed that H_2_L has emissive properties in DMSO, showing a band centred at λ = 600 nm when excited at λ = 475 nm (Figure S1b). This property vanishes in 60% (*v*/*v*) DMSO/H_2_O solvent mixtures.

### 3.2. Metal Coordination

Following our previous studies with Zn(II) [23], we decided to evaluate the ability of the ligand to coordinate other transition metal ions with different properties, such as oxidation state and preferential coordination geometry. Cu(II), Ni(II), Ru(III), V(IV)O and Fe(III) ions were selected, and synthetic procedures were performed to obtain complexes with metal-to-ligand (M:L) stoichiometries of 1:1 and 1:2. Nevertheless, for some metal ions (V, Ru and Fe), only one type of stoichiometry was obtained based on the characterisation data. Interestingly, from a Ru(III) precursor we obtained a Ru(II) complex. Overall, the elemental analysis and mass spectrometry performed for the complexes are in good agreement with the following structural formulae: [Cu^II^(HL)Cl], [Cu^II^(HL)_2_], [Ni^II^(HL)(acetate)], [Ni^II^(HL)_2_], [Ru^II^(HL)Cl(DMSO)], [V^IV^O(HL)_2_] and [Fe^III^(HL)_2_Cl(H_2_O)]. Their structure and spectroscopic properties will be described next.

First, the presence of the chloride ion in [Cu^II^(HL)Cl], [Fe^III^(HL)_2_Cl(H_2_O)] and [Ru^II^(HL)Cl(DMSO)] complexes was confirmed with FTIR spectroscopy (in CsI pellets) by the presence of low energy vibrations, recorded at 312, 320 and 331 cm^−1^, respectively (see Figure S3). The FTIR of [VO(HL)_2_] (in KBr pellet, Figure S4) presents a medium sharp band at 983 cm^−1^ corresponding to the characteristic V = O stretching vibration, confirming the presence of an oxidovanadium centre [43]. The V(IV)O and Fe(III) complexes’ FTIR spectra (Figure S4) show broad bands at 3400 cm^−1^, in a clear indication of the deprotonation of the phenol group upon coordination, while little changes are observed in the region 1620–1440 cm^−1^, dismissing the coordination of the imine moiety in these complexes.

A broad band centred at 3400 cm^−1^ is also present in [Cu^II^(HL)Cl] and [Ni^II^(HL)(acetate)] spectra, supporting the coordination through the O-phenolate of the hydroxyquinoline moiety (see Figures S5 and S6). The absence of NMR spectra for the d^8^ Ni(II) 1:1 complex reveals an octahedral geometry around this metal centre, with an electronic distribution that yields two unpaired electrons. In contrast, the FTIR spectra of [Cu(HL)_2_], [Ni(HL)_2_] and [Ru(HL)Cl(DMSO)] (see also Figure S7) display sharp bands at 3411 cm^−1^, indicating that in these complexes the phenol group is protonated and not involved in the metal coordination. Substantial changes in the 1620–1144 cm^−1^ region point to the coordination of imine and benzothiazole nitrogen atoms in the first two, while the disappearance of the signal at 520 cm^−1^ in the Ru complex points to 8-hydroxyquinolinate coordination. This coordination sphere is further corroborated by the ^1^H NMR spectrum of the [Ni(HL)_2_] complex in DMSO_*d*_6_ (Figure S8), in which the presence of the OH signal can be observed at the same chemical shift as found for the ligand precursor, and as in [Ru(HL)Cl(DMSO)] (Figure S9). The FTIR spectrum of [Ru(HL)Cl(DMSO)] (Figure S7) does not show the NH band, indicating the deprotonation of the ligand at this moiety. A strong band at 1019 cm^−1^, corresponding to the rocking (ρ) of the CH_3_ groups, and another at 1108 cm^−1^ [ν(S = O)] confirm the presence of a dimethyl sulfoxide molecule in this complex [24]. The deprotonated form of the ligand in [Ru(HL)Cl(DMSO)] is further confirmed in the ^1^H NMR spectrum (Figure S9) with the absence of the NH signal, while a broad peak at 10 ppm shows the presence of OH. The observation of a diamagnetic character for the Ru-complex indicates that reduction took place during the synthetic procedure and from a Ru(III) precursor, a Ru(II) species was obtained. This is not uncommon since Ru has a low reduction potential and triethylamine, the base used to undertake ligand deprotonation, might have been oxidised [44]. This has been previously observed with other Ru(III) complexes [45].

The Cu(II) and V(IV)O complexes, being paramagnetic, were also analysed by electron paramagnetic resonance (EPR, see Figures S10 and S11) spectroscopy and the spin Hamiltonian parameters were obtained by spectral simulation [46], Table 1. In both Cu(II) complexes, the frozen solution X-band EPR spectra in DMF are axial, presenting g_z_ > g_x,y_ > 2.0, which suggests the presence of a d_x_^2^_−y_^2^ ground state in copper(II) located in square-based geometries [47]. The [VO(HL)_2_] complex, dissolved in methanol, also shows axial EPR spectra with a hyperfine pattern typical of V^IV^O-complexes and consistent with the presence of monomeric V^IV^O-bound species with a $d_{xy}^{1}$ ground-state.

Considering the values obtained for g_z_ and |A_z_| in both Cu(II) complexes, and according to the empirical plot proposed by Peisach and Blomberg, N_2_O_2_, N_4_ or N_3_O binding arrangements are expected [48]. This suggests that in DMF solution the chloride ion is not coordinated to Cu in the 1:1 complex and acts as a counter ion (binding set N_3_O). For [Cu(HL)_2_], both N_2_O_2_ and N_4_ are possible donor sets. Bearing in mind the FTIR data, N_4_ is proposed. Moreover, the values obtained for the g_z_/|A_z_| ratio are higher than 135 cm, indicating the presence of tetrahedral distortions from square planar-based coordination geometries [49], due to steric hindrance, imposed by the ligand planarity.

For the oxidovanadium(IV) complex, the empirical Chasteen additivity rule [50] [|A_z_^est^| = Ʃ|A_zi_| (i = 1–4), with |A_zi_| being the contribution of each of the four equatorial donor atoms] can be used. The proposed equatorial coordination is [2×phenolate-O_−_, 1×quinoline-N, 1×OH_2_], for which the |A_z_^est^| = 163.2 × 10_−4_ cm_−1_. These values are in good agreement with the ones reported by G. Scalese et al., in particular with their compound [V^IV^O(L5H_−1_)_2_] [51], which involves the coordination of two 2-aldehyde-8-hydroxyquinoline molecules, the precursor used for the synthesis of H_2_L [52].

The UV-vis absorption spectra of the complexes in DMSO (see Figures S12–S14) show the characteristic intraligand π→π* transitions, in roughly the same wavelengths, with new bands appearing at lower energy, corresponding to charge transfer mechanisms between the ligand and the metal centres—see Table 2. Due to low solubility in common solvents, no d-d bands could be observed and assigned. All complexes show low solubility and stability in PBS buffered solutions, and the Fe(III) complex is almost insoluble. Spectral changes suggest precipitation, except for the V(IV)O complex for which oxidation of the metal centre likely takes place. To corroborate this, a small amount of [VO(HL)_2_] was mixed with 2 equivalents of KOH and left stirring under air in methanol at r. t. over two days. An orange solid was collected and analysed by ^51^V NMR spectroscopy showing the presence of a resonance at δ= −527 ppm (standard VOCl_3_ at δ = 0 ppm), in agreement with the formation of a [V^V^O_2_L] type complex. Its chemical shift is in the same range as found for similar complexes [53].

**Figure 3:**
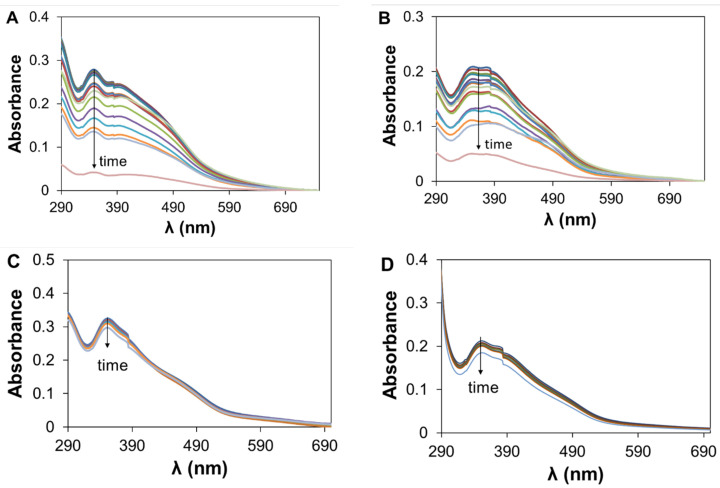
Aqueous stability tests with Cu(II) complexes 1:1 (**A**,**C**) and 2:1 (**B**,**D**) in the absence (**A**,**B**) and in the presence of equimolar amounts of BSA (**C**,**D**). The complexes’ concentration was ca. 25 µM with <1% DMSO and the measurements were done up to 24 h.

In general, the spectral intensity in buffered aqueous solutions strongly decreases within 24 h (Figure 3A,B and Figure S15); however, the presence of BSA clearly avoids this problem and allows the recording of almost unchanged UV-vis spectra for at least 24 h—Figure 3C,D and Figure S15. This is important since mammal cells incubation media contains ca. 40 μM of BSA added in FBS and will help to protect the complexes from precipitation.

**Scheme 1:**
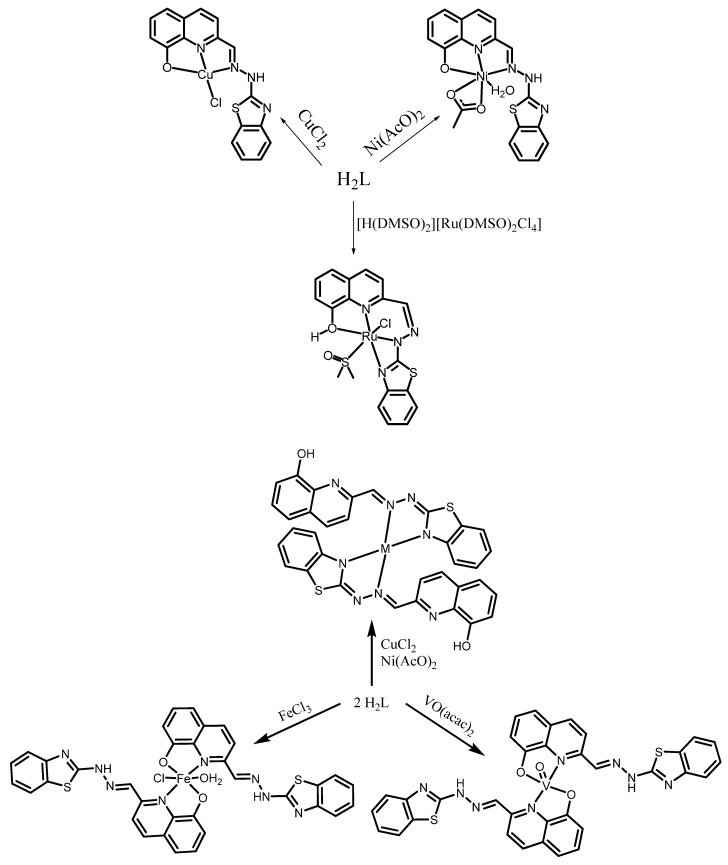
Schematic representation of 1:1 copper(II), nickel(II) and ruthenium(II) and 2:1 copper, nickel, vanadium and iron complexes.

Overall, the analytic and spectroscopic data suggest that the ligand adopts different coordination modes with different metal ions and stoichiometries. In the 1:1 stoichiometry, the ligand is probably tridentate and acts as monoanionic through the hydroxyl group (Cu^II^ and Ni^II^) or through the hydrazinic moiety (Ru^II^). For the 1:2 stoichiometry, the ligand is bidentate and monoanionic, but coordination involves either the hydroxyquinolinate moieties (V^IV^O and Fe^III^) or the imine and benzothiazolate nitrogen atoms (Cu^II^ and Ni^II^), while keeping the phenol protonated. Considering all the experimental evidence, structural formulae for the complexes are proposed in Scheme 1.

### 3.3. Light Sensitivity of H_2_L and Its Ru-Complex

**Figure 4:**
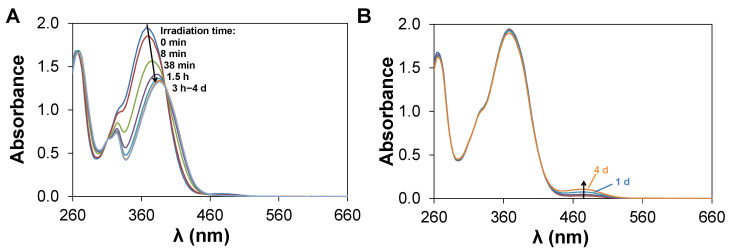
Time-dependent UV-vis spectra of the ligand in DMSO. (**A**) Irradiated continuously with diffuse light. (**B**) Protected from light. [*c*_ligand_ = 122 μM; *ℓ* = 2 mm; *T* = 25.0 °C].

The light sensitivity of H_2_L and its Ru-complex (as model metal compound) were tested by UV-vis spectrophotometry to further understand their behaviour. Samples were dissolved in DMSO and stored in the dark, while one portion was irradiated continuously by diffuse light. Both the ligand and the complex showed strong light sensitivity, which resulted in substantial changes in the UV-vis spectra. A relatively fast change is observed for the ligand in the first 1.5 h, and after that the spectrum has only minor changes (Figure 4A). In dark, only a small change is visible, accompanied by the very slow development of a new band with *λ*_max_ = 474 nm (Figure 4B). Figure S16 depicts the spectral changes visible in Figure 4B. The inset shows the change of the absorbance values at λ_max_ = 474 nm as a function of time. The changes follow a saturation curve, however, this reaction did not reach the final stage during the monitored period (4 days). In the ligand, this may be due to a photoisomerisation process of the imine bond (Z/E isomers), which is quite common in hydrazones [54]), but not in the Ru-complex, unless what is being observed in the complex solution is due to unbound ligand molecules. The Ru-complex showed identical changes during irradiation (Figure S17), while the samples in dark showed no changes during the 4 days of monitoring. 

^1^H NMR spectra were also recorded for samples protected from light and irradiated. Figure S18 shows that in the samples protected from light a slight change is visible, namely a singlet peak that goes from 8.34 ppm to 5.77 ppm. In the chemical shift range 3.2−4.9 ppm, a new peak set is developed (not shown) which can be due to complexation with metal ion traces. A parallel sample was irradiated with diffuse light, and after 2.5 days it showed a clear difference from the light protected sample, since a new peak set is observed, which is not similar to the spectrum of 2-hydrazinobenzothiazole, a possible decomposition product.

**Figure 5:**
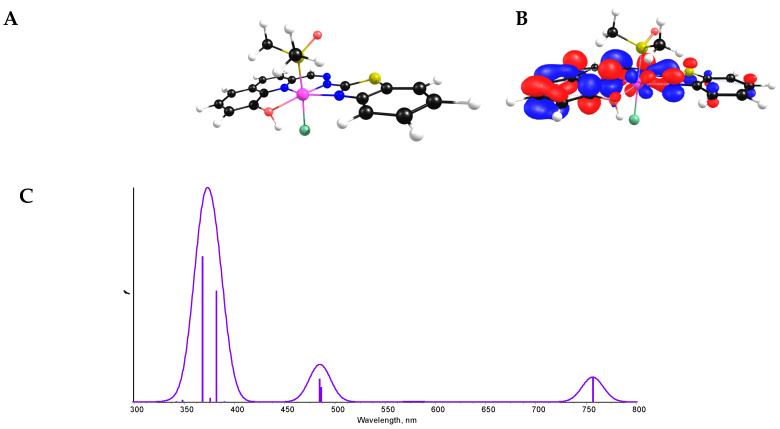
(**A**) DFT structure or the Ru complex, (**B**) LUMO orbital of the Ru complex and (**C**) corresponding absorption spectra.

DFT calculations in the Ru complex predict a tetra-bonded ligand with the hydroxyl hydrogen making an intramolecular H-bond to the chloride atom, Figure 5A. The absorption spectrum depicts a low energy band at 760 nm, which is observed in the measured spectrum at 770 nm (Figure S14). This is the HOMO-LUMO transition of MLCT character and is antibonding towards the hydroxyquinoline moiety, Figure 5B, between metal and ligand leading to the HL^-^ un-coordination upon irradiation explaining the behaviour described above.

### 3.4. Lipophilicity of the Title Ligand and Its Ru Complex

The distribution coefficients (*D*_7.4_) for H_2_L and the Ru-complex (as model complex) were determined using HEPES buffer (pH = 7.4) for the aqueous phase. Distribution was measured in the absence and presence of 100 mM KCl. The latter condition mimics the chloride ion concentration of the blood plasma. Due to the predicted high lipophilicity of these compounds at neutral pH, an aqueous phase:*n*-octanol phase = 9:1 volume ratio was used. Samples were dissolved in *n*-octanol, and after partitioning and separation the UV-vis spectra for both phases were recorded. Both the ligand and the Ru-complex showed high lipophilicity and the presence of chloride ion had only a slight effect on the coefficients, as shown in Table 3.

While the UV-vis spectrum of ligand remained unchanged after partitioning, the complex suffered changes in the wavelength range λ > 400 nm (Figure S19), which indicates changes in the coordination sphere. This may be the result of the possible coordination of water or additional chloride ions. 

### 3.5. Determination of Proton Dissociation Constants

**Figure 6:**
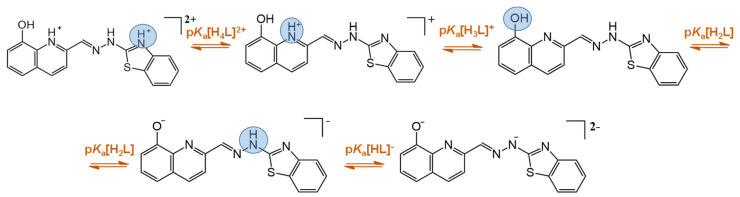
The suggested deprotonation steps of the ligand H_2_L.

The p*K*_a_ values of bioactive compounds are crucial factors influencing the protonation state under physiological conditions and the solubility at a given pH, having therefore a great impact on the pharmacokinetic properties. H_2_L has four dissociable protons, namely the protonated benzothiazole nitrogen (NH^+^_btia_), the quinolinium nitrogen (NH^+^_quin_), the hydroxyl group (OH), and the hydrazinic nitrogen (NH^+^_hydr_) (Figure 6). The p*K*_a_ values predicted by the Marvin software [55] suggest two overlapping deprotonation processes in the acidic pH range and two overlapping processes in the basic pH range (Table 4).

**Figure 7:**
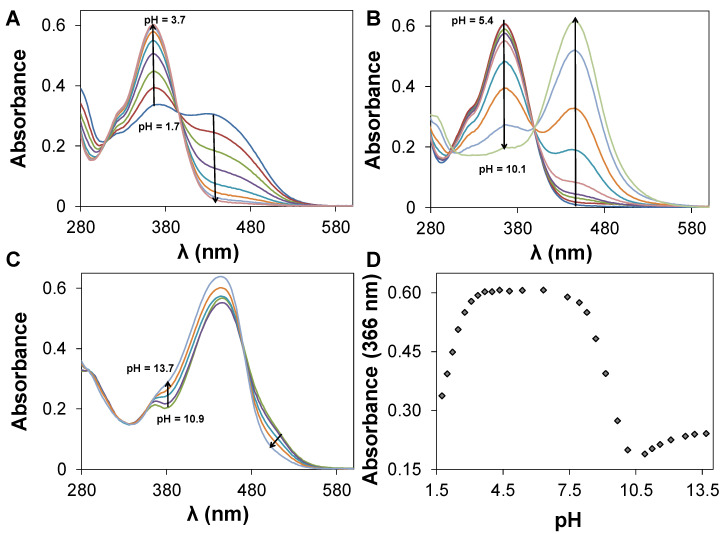
UV-vis spectra of the ligand recorded at various pH values in 60% (*v*/*v*) DMSO/H_2_O mixture, showing: (**A**) the first deprotonation step; (**B**) the second deprotonation step; (**C**) the third deprotonation step. (**D**) Absorbance changes at 366 nm as a function of pH, showing all visible deprotonation steps of the ligand in 60% (*v*/*v*) DMSO/H_2_O mixture. [*c*_ligand_ = 4.86 μM; *I* = 0.10 M (KCl); *ℓ* = 5 cm; *T* = 25.0 °C].

Titrations were performed first in 30% (*v*/*v*) DMSO/H_2_O mixture due to the limited water solubility of the ligand. The sample was protected from light and, for a given portion of the sample, the UV-vis spectrum was recorded only once. Despite the high DMSO content and low concentration (5 μM), the ligand precipitated in a wide pH range (3.8−11.0, Figure S20), indicating that a neutral species is dominating under these conditions. Following this experiment, the titration was repeated in 60% (*v*/*v*) DMSO/H_2_O mixture (Figure 7), and no precipitation was found in the whole pH range (1.73−13.67) under this condition. However, only three deprotonation steps were observed, most probably because one of the deprotonation steps was already completed at the starting pH. The determined p*K*_a_ values are listed in Table 4.

From these results, it is still unclear to which functional group belongs the first deprotonation process. To elucidate the p*K*_a_ assignment, 2-hydrazinobenzothiazole (considered as a fragment of the title ligand) was titrated under similar conditions to monitor the spectral changes during the deprotonation of the benzothiazole moiety. All deprotonation steps were accompanied by a bathochromic shift for this fragment, while the first deprotonation resulted in a hypochromic shift in the UV-vis spectrum of the title ligand. Based on this observation, the benzothiazole moiety was already deprotonated at the starting pH and the first process is considered the deprotonation of NH^+^_quin_ (p*K*_a_ = 1.99 ± 0.03).

The spectrophotometric titration of the ruthenium complex was performed in the same way with samples carefully protected from light. Surprisingly, all spectral changes are identical with those of the ligand. Most probably the complex dissociates in the 60% (*v*/*v*) DMSO/H_2_O mixture, under the conditions used. To confirm this, ^1^H NMR spectra were recorded for the ligand and the complex in DMSO_*d*_6_ and in 60% (*v*/*v*) DMSO_*d*_6_/aqueous buffer (pH = 7.4, 10 mM HEPES) mixture. While the complex has a different spectrum from the ligand in DMSO_*d*_6_ (Figure S21a), in buffered aqueous solution as co-solvent very similar spectra are recorded (Figure S21b). Most of the complex is dissociated in the solvent mixture, which can be responsible for the similarity in the UV-vis titration.

### 3.6. Albumin Binding

Spectrofluorimetric studies show that mixtures of all compounds and BSA display significant fluorescence quenching in a concentration-dependent way. However, due to the light sensitivity of the samples, these experiments were not quantitatively analysed. The intense light of the fluorimeter can cause decomposition of the compounds, even in experiments performed in the dark. Thus, we cannot exclude that the quenching is due to binding of side-products to albumin, instead of the original ligand or complex. Moreover, circular dichroism (CD) titrations showed the development of high intensity induced CD bands centred at ca. 360 nm, particularly for H_2_L, [Ni(HL)(acetate)], [Ru(HL)Cl(DMSO)] and [VO(HL)_2_]. Since under the conditions used [*c_compoud_* between 5 and 70 μM)] no metal centred bands are observed, we cannot exclude that these are due to the ligand or its side-products binding the protein. Nonetheless, spectroscopic titrations seem to suggest that the compounds can bind albumin, as already indicated by the increased stability of the compounds in the presence of this protein.

### 3.7. MTT Assay

Several metal-based complexes were synthesised using the Schiff base derived from 8-hydroxyquinoline and benzothiazole, aiming to obtain new metallodrugs for anticancer therapy. Thus, to evaluate the antiproliferative properties of the newly synthetised metal complexes, a preliminary screening was performed using human (HCT-116 and A375) and murine (CT-26 and B16F10) colon cancer and melanoma cell lines, respectively. The human keratinocyte cell line, HaCaT, was also included in this in vitro study aiming to evaluate the specificity of the new compounds against tumour cells. Complexes were evaluated at concentrations of 20 and 40 μM and the obtained results are depicted in Figure S22. The ligand, H_2_L, as well as the molecules used to synthetise it was also tested. While the benzothiazole precursor (HzBzTz) presented no effect, the aldehyde (HQ2CHO) showed cytotoxicity against the human melanoma and colon cancer cell lines. Regarding the ligand, a high cytotoxicity was observed for all tested cell lines (Figure S23 and Table S1). Similar results have been also described for other different Schiff bases [56,57]. 

Overall, most complexes exhibited antiproliferative activity towards all cell lines, with the exception of [Fe(HL)_2_Cl(H_2_O)], which showed no cytotoxic effect in the tested concentrations. Among all screened compounds, [Ru(HL)Cl(DMSO)] and [Ni(HL)(acetate)] were selected for further in vitro studies based on their higher selectivity towards colon cancer cell lines in comparison to HaCaT (Figure S22).

**Figure 8:**
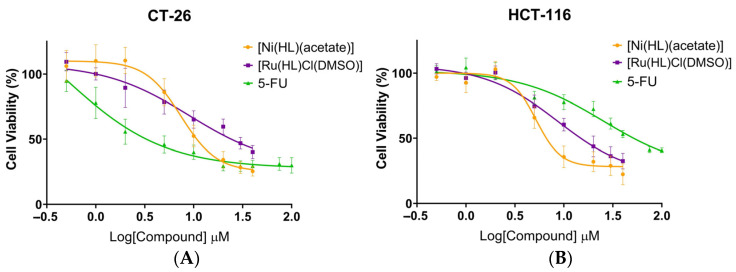
In vitro antiproliferative activity of [Ni(HL)(acetate)], [Ru(HL)Cl(DMSO)] and 5-FU against CT-26 (**A**) and HCT-116 (**B**) colon cancer cells after 48 h incubation.

MTT assay was performed for the selected Ni and Ru complexes using a wide range of concentrations from 0.5 to 40 μM. The respective dose-response curves are shown in Figure 8A,B and the calculated IC_50_ values are presented in Table 5.

Both complexes demonstrated notable antiproliferative activity in murine and human colon cancer cell lines, with [Ni(HL)(acetate)] proving to be more effective than [Ru(HL)Cl(DMSO)]. Importantly, in HCT-116 cells, both complexes displayed higher cytotoxicity than the clinically approved 5-FU. 

In the literature, several other nickel complexes have also exhibited antiproliferative activity against colon cancer cell lines. For example, two Ni-Schiff base complexes exhibited IC_50_ values of 7.9 [58] and 26.5 μM [59] towards HCT-116 cells, after 48 h incubation. Additionally, three other Ni complexes containing thiosemicarbazones were incubated with HCT-116 cells for 72 h, displaying IC_50_ values between 11.7 and 18 μM [56], the same range as our nickel complex after a shorter (48 h) incubation period. In fact, some of the nickel complexes, reported by Heng and co-workers, were equally or less cytotoxic when compared with their respective ligand, suggesting that the observed antitumour activity may not derive from complexation with nickel [56].

Likewise, many ruthenium complexes have been described in the literature as potential compounds against colon cancer. When tested against CT-26 cells, two ruthenium compounds demonstrated IC_50_ values of 50.9 and 148 μM, after a 72 h incubation time, a much lower cytotoxic effect compared to our ruthenium complex [60]. Many other complexes of this metal have been tested against HCT-116 cells. For example, Kubanik et. al. evaluated a variety of Ru complexes with the same 8-hydroxyquinoline scaffold but with different substituents. Almost all showed high cytotoxic activity with IC_50_ values ranging from 1.98 to 14.6 μM [61]. Furthermore, in a study conducted by Xu and co-workers [62], several Ru complexes have also exhibited IC_50_ values in the low micromolar concentration range against colorectal, liver, lung and cervical carcinoma cell lines, being more potent than the clinically approved cisplatin [62]. 

On the other hand, several other studies reported lower potency of ruthenium compounds against HCT-116 cells (IC_50_ values from 22 to > 100 μM) [58,62] compared to our Ru complex, [Ru(HL)Cl(DMSO)]. Moreover, two 8-hydroxyquinoline Ru complexes with a similar structure to [Ru(HL)Cl(DMSO)] exhibited up to 18-fold lower antiproliferative activity [63]. In another study, a ruthenium-based 5-FU complex was synthetised and tested against a large panel of different cell lines. In most cases, this hybrid complex demonstrated superior cytotoxicity compared to 5-FU alone and, a 3-fold increase in potency was observed towards HCT-116 cells [64]. 

Noteworthy, NAMI-A was the first Ru complex to enter clinical trials in patients with non-small cell lung cancer [65] and solid tumours [66]. Curiously, previous in vitro studies showed minimal cytotoxicity against cancer cells [67] exhibiting a 1000-fold lower activity than cisplatin [68]. Nevertheless, in vivo experiments demonstrated NAMI-A antimetastatic potential and safety [69]. 

### 3.8. Guava ViaCount Assay

**Figure 9:**
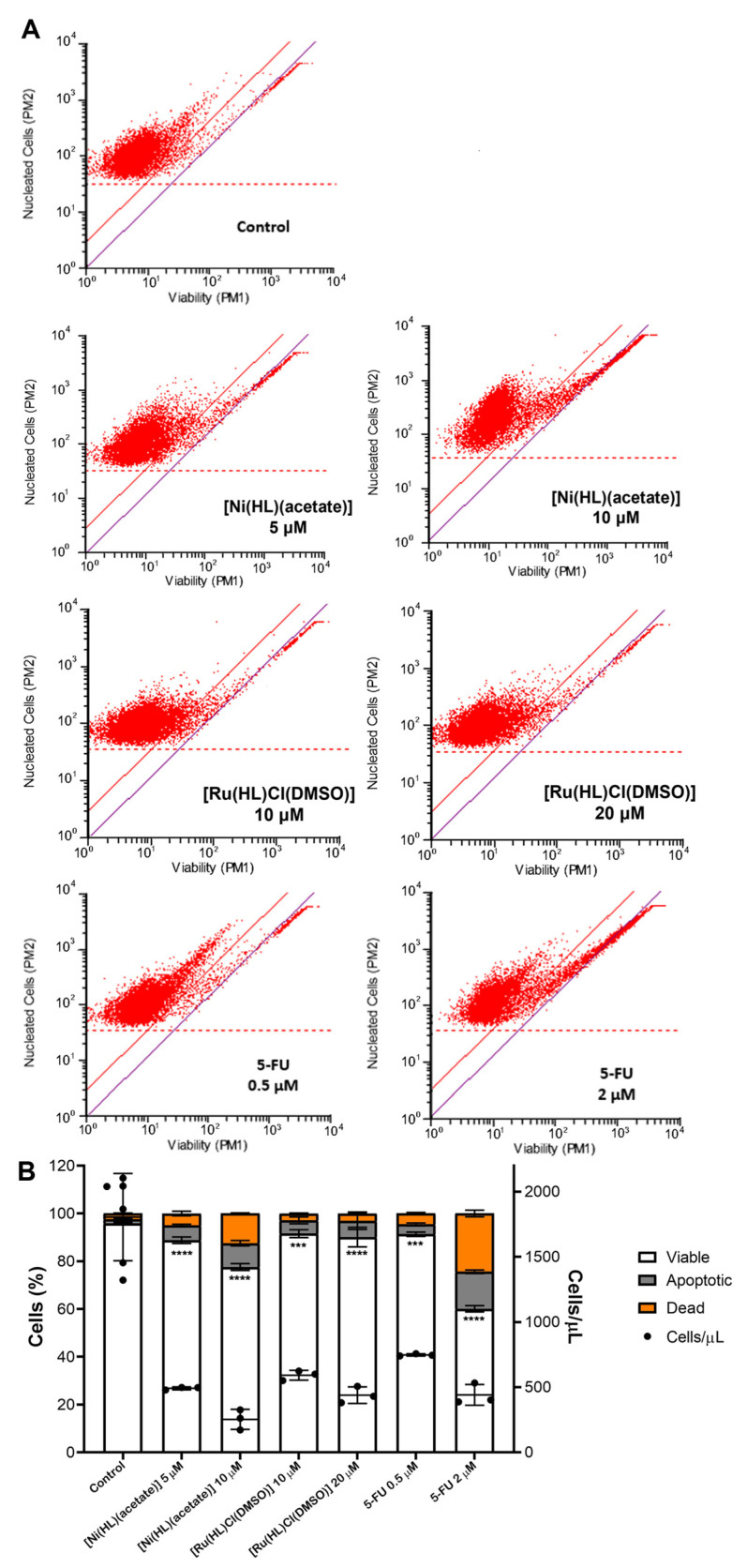
Evaluation of [Ni(HL)(acetate)], [Ru(HL)Cl(DMSO)] and 5-FU effect on CT-26 cell viability by Guava ViaCount. (**A**) Cell populations obtained by Guava ViaCount flow cytometry analysis after 48 h incubation with different concentrations of each compound. (**B**) Percentage of viable, apoptotic and dead cells, as well as number of cells/μL. Data are expressed as mean ± SD of two independent experiments. *** *p* < 0.001, **** *p* < 0.0001 vs. control. Statistical analysis was performed by two-way ANOVA with Dunnett’s test.

To corroborate data from MTT assays, the analysis of cell viability by Guava ViaCount flow cytometry was also performed for the murine colon cancer cell line, CT-26 (Figure 9A,B). A concentration-dependent decrease in cell viability was observed for the tested compounds, being in-line with the data described in Section 3.7. A higher percentage of viable cells was observed for the tested compounds in comparison to data from MTT assay. 

Importantly, when compared to the negative control, a much lower cell concentration (three-fold) was observed following incubation with the metal-based complexes under study (Figure 9B). While MTT assay evaluates the mitochondrial activity of adherent cells, in Guava Via count all cells are analysed using two DNA binding dyes [33,34]. Nevertheless, these results confirm the inhibition of cancer cell proliferation and induction of cell death by the Ni and Ru complexes. For 5-FU, a higher percentage of dead and apoptotic cells was observed. These results might suggest an advantage of our complexes in terms of safety as their action was predominantly to halt cell proliferation, unlike 5-FU, which considerably induced cell death.

### 3.9. Cell Cycle Analysis

**Figure 10:**
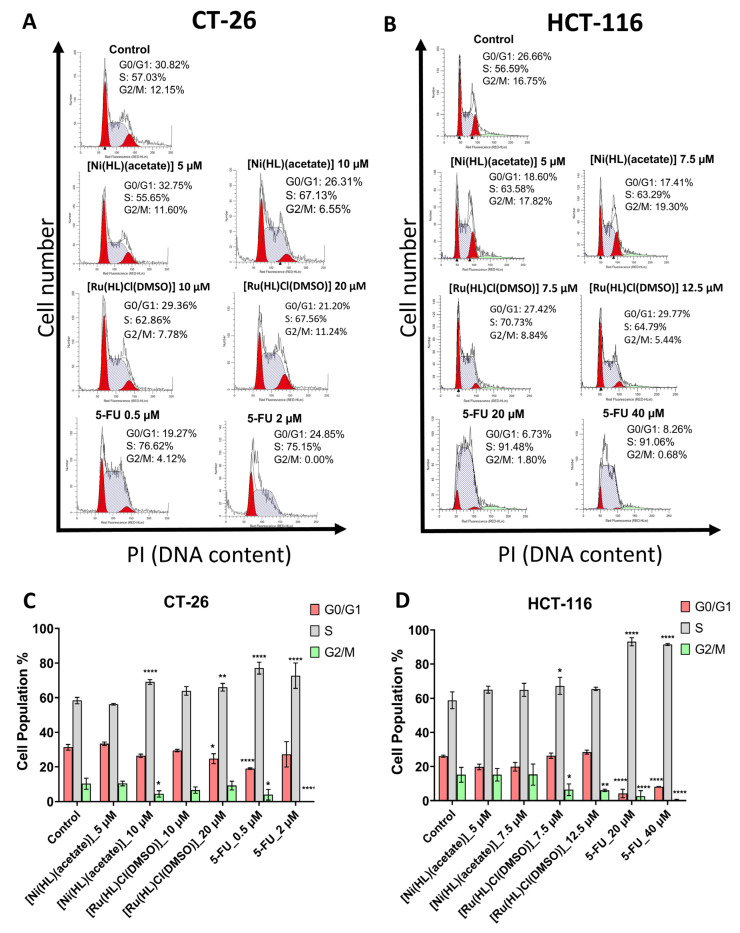
Representative histograms of CT-26 (**A**) and HCT-116 (**B**) cell cycle analysis. (**C**) Distribution of CT-26 cell population in % at different cell cycle phases after incubation with complete medium (control) or with [Ni(HL)(acetate)] at 5 and 10 μM, [Ru(HL)Cl(DMSO)] at 10 and 20 μM and 5-FU at 0.5 and 2 µM, for 24 h. (**D**) Distribution of HCT-116 cell population, in %, at different cell cycle phases after incubation with complete medium (control) or with [Ni(HL)(acetate)] at 5 and 7.5 μM, [Ru(HL)Cl(DMSO)] at 7.5 and 12.5 μM and 5-FU at 20 and 40 µM, for 24 h. Data are expressed as mean ± SD of two independent experiments. * *p* < 0.05, ** *p* < 0.01, **** *p* < 0.0001 vs. control.

One of the hallmarks of cancer is the rapid overgrowth of malignant cells, which mainly happens due to a dysfunction in their cell cycle regulation [56,70,71]. The induction of cell cycle arrest is, therefore, an important anticancer mechanism of action which helps reduce the uncontrolled cell proliferation. This mechanism is exploited by many anticancer drugs, including the positive control used in this study, 5-FU [56,58,70]. In this sense, to better understand how [Ni(HL)(acetate)] and [Ru(HL)Cl(DMSO)] exert their antitumour effect, cell cycle progression was evaluated in CT-26 and HCT-116 cell lines (Figure 10). To accomplish this, cells were incubated for 24 h with [Ni(HL)(acetate)], [Ru(HL)Cl(DMSO)] and 5-FU at sub-toxic concentrations (below the IC_50_) to avoid excessive cytotoxicity, thus ensuring the quality of obtained results [71,72].

[Ni(HL)(acetate)] arrested cell cycle at S phase in murine CT-26 cells at a concentration close to its IC_50_ value (Figure 10A,C); however, no such effect was observed in the human HCT-116 cells at the tested concentrations (Figure 10B,D). In the literature, different nickel complexes also showed the capacity to arrest the cell cycle of leukocytes [73] and human gastric carcinoma cells [74] at S phase. Furthermore, in MCF7 human breast cancer cells, another nickel complex triggered cell cycle arrest at S phase and enhanced DNA fragmentation, ultimately inducing cell apoptosis [59]. On the other hand, two other Schiff base ligands, along with their respective nickel complexes, arrested cell cycle at G0/G1 phase, in HCT-116 cells [56]. 

For [Ru(HL)Cl(DMSO)] a cell cycle arrest at S phase in both murine and human colon cancer cell lines was achieved. The action of other ruthenium complexes on cell cycle progression has also been widely described in the literature. A Ru triphenylphosphine complex [59] and a Ru terpyridine complex [60] demonstrated the ability to arrest cell cycle at G2/M phase in HCT-116 cells. Additionally, a bis-pyrazolylpyridine Ru(III) complex was shown to arrest CT-26 cell cycle at G0/G1 phase. This mechanism of action is useful, since it stops cancer cell proliferation; however, acting at early phases of the cell cycle may allow cells to repair the damage caused by the anticancer agent [68]. Human HT-29 colon cancer cell lines incubated with a Ru quercetin complex [75] and a Ru phloretin complex [76], arrested cell cycle at G0/G1 and G2/M phases, respectively. The ruthenium 5-FU complex, previously mentioned in Section 3.7, did not arrest the cell cycle of HCT-116 cells at any phase, opposed to 5-FU alone that increased the number of cells in S phase. These results demonstrate the impact that both metals and ligands may exert in the biological activity of compounds [64]. 

In the present work, 5-FU was used as a positive control group. This clinically approved molecule is an antimetabolite anticancer drug that mainly impairs DNA replication during S phase [64,77,78], inhibiting cell cycle progression and inducing cell apoptosis [77,78]. In vitro studies performed by Afrin et al. and Yang et al. on HCT-116 cells have showed that 5-FU, at concentrations around 20 μM, arrested cell cycle at S phase, [79,80] thus validating our results. 

Overall, it is well-known the variety of alternative mechanisms of action by which metal complexes may exert their biological or pharmacological activity, besides impacting cell cycle [4,8,67]. Various nickel complexes have shown to act through the formation of ROS species and inducing mitochondrial-mediated apoptosis [56,74,81,82] or by activating the tyrosine kinase proapoptotic pathway [58]. In addition, other ruthenium complexes have also demonstrated to exert activity through the apoptotic pathway via cleaved PARP, caspase-3 and caspase-9 [64,83]; lysosome-mediated apoptosis [62]; necrosis [60]; and possibly inhibiting DNA replication by binding to DNA strands [59].

### 3.10. Migration Assay

**Figure 11:**
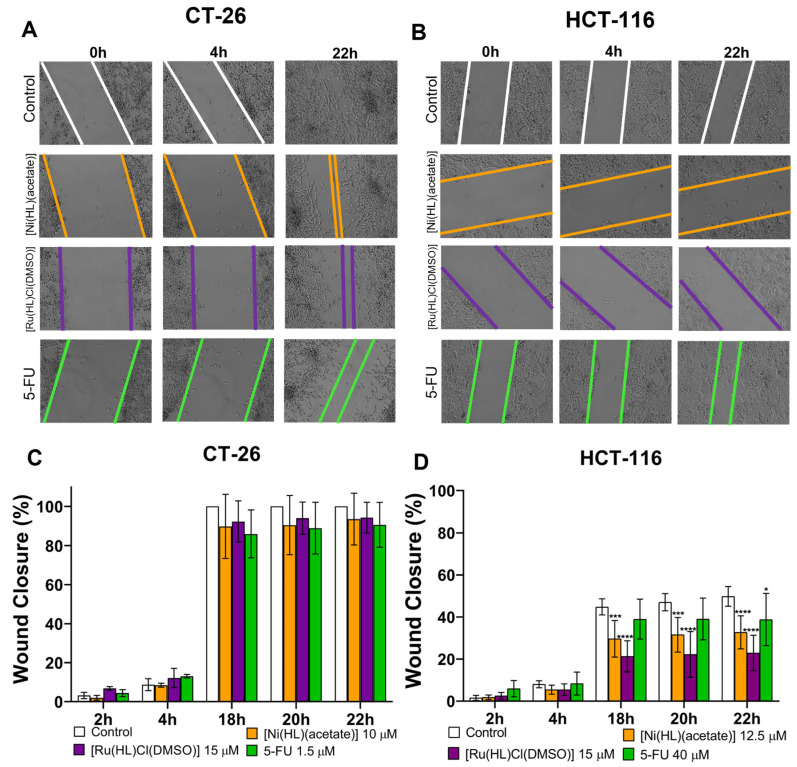
Representative images of CT-26 (**A**) and HCT-116 (**B**) cell migration in the absence (negative control) or presence of [Ni(HL)(acetate)], [Ru(HL)Cl(DMSO)] and 5-FU (positive control) at fixed time periods. Wound closure progression (%) of CT-26 (**C**) and HCT-116 (**D**) cells. Data are expressed as mean ± SD of two independent experiments. * *p* < 0.05, *** *p* < 0.001, **** *p* < 0.0001 vs. control. Statistical analysis was performed by two-way ANOVA with Dunnett’s test.

Cancer metastasis occurs in latter stages of the disease, involving the spreading of cancer cells from the primary tumour site to secondary sites, and represents a major factor in cancer-related deaths [84,85]. Cell migration plays a pivotal role in cancer metastasization and, therefore, it is important to investigate the potential effect of novel compounds in this cellular process [36,62,67,81]. In this regard, the antimetastatic potential of [Ni(HL)(acetate)] and [Ru(HL)Cl(DMSO)] was evaluated through a wound-healing assay in CT-26 and HCT-116 cell lines. To accomplish this, cells were incubated with [Ni(HL)(acetate)], [Ru(HL)Cl(DMSO)] and 5-FU at concentrations close to the IC_50_ value of each compound [81]. Cell migration was monitored over time after wound scratch, as depicted in Figure 11A–D. In CT-26 cells, no significant inhibitory effect was observed for any compound in comparison to the negative control. However, in HCT-116 cells, a significant inhibition of cell migration was observed for both metal complexes. Wound closure in cells treated with [Ni(HL)(acetate)] and [Ru(HL)Cl(DMSO)] reached a maximum of 32% and 22%, at 22 h, respectively. Comparatively, the control and 5-FU treated cells exhibited maximum wound closure values of 50% and 39%, at 22 h, respectively. 

Some nickel complexes have shown high suppression of cell migration in lung carcinoma cell lines, possibly due to the silencing of genes related to epithelial-mesenchymal transition [86]. In a study conducted by Qi and co-workers, a nickel complex also inhibited cell migration of hepatoma carcinoma cells [87]. Similarly, in the literature, various examples of ruthenium complexes demonstrating antimigratory effects can be found. A study performed by Xu et al. showed that CT-26 cells treated with a ruthenium complex displayed a pronounced inhibitory effect likely due to downregulation of the cell migration-related proteins metalloproteinase-2 and metalloproteinase-9 [62]. Additionally, as aforementioned, the ruthenium complex NAMI-A has entered clinical trials due to its specific antimetastatic activity [67]. This compound has also demonstrated an inhibitory effect in reducing cell adhesion and migration in HCT-116 cell line. This complex impaired the interaction between the cells and fibronectin and collagen I, two extracellular proteins closely related to cellular migration in the liver, an organ where colon cancer often metastasizes [67].

## 4. Conclusions

In this study, novel metal complexes were synthetised using a Schiff base derived from 8-hydroxyquinoline and benzothiazole. Seven new complexes were obtained and characterised by analytical and spectroscopic techniques, with the following stoichiometries being assigned: [Cu(HL)Cl], [Cu(HL)_2_], [Ni(HL)(acetate)], [Ni(HL)_2_], [Ru(HL)Cl(DMSO)], [VO(HL)_2_] and [Fe(HL)_2_Cl(H_2_O)]. These reveal the versatility of the ligand upon metal coordination since different binding modes are proposed for each complex based on their spectroscopic signatures. Ligand proton dissociation constants were determined by spectrophotometry in 60%DMSO:40%H_2_O, yielding the following sequence of deprotonation: NH^+^_benzothiazole_, NH^+^_quinoline_, OH and NH_hydrazone_. The compounds showed high lipophilicity and light sensitivity.

After physicochemical characterisation of the obtained compounds, two were selected for further in vitro assays, [Ni(HL)(acetate)] and [Ru(HL)Cl(DMSO)]. These two metal complexes showed notable antiproliferative effect in both murine and human colon cancer cell lines, as demonstrated by MTT and confirmed by Guava ViaCount assay. In the human cell line, HCT-116, both compounds demonstrated superior antitumour activity when compared to 5-FU, a first-line drug currently used in colorectal cancer patients, highlighting their potential as anticancer agents. In terms of cell cycle progression, both [Ni(HL)(acetate)] and [Ru(HL)Cl(DMSO)] arrested cell cycle of murine CT-26 cells in the S phase in a concentration-dependent manner. In turn, for the human HCT-116 cells, the same effect was only observed for [Ru(HL)Cl(DMSO)]. This mechanism of action is in accordance with the positive control used in this study, 5-FU, which interferes with DNA replication. In addition, cell migration of HCT-116 was significantly impaired by both complexes. 

Overall, these promising results emphasise the therapeutic potential of these nickel and ruthenium complexes as alternative and effective therapies against colorectal cancer. 

## Figures and Tables

**Table 1:** Spin Hamiltonian parameters determined by simulation of spectra measured at ca. 100 K [46]. The copper complexes were dissolved in DMF (≈3 mM), while for vanadium the solvent was methanol (≈1 mM).

Complex	g_z_	g_x,y_	|A_x,y_| (×10^−4^ cm^−1^)	|A_z_| (×10^−4^ cm^−1^)	g_z_/|A_z_| (cm)
[Cu(HL)Cl]	2.268	2.071	26.4	163.1	139.1
[Cu(HL)_2_]	2.279	2.081	31.0	161.1	141.5
[VO(HL)_2_]	1.958	1.985	57.6	163.8	-------

**Table 2:** UV-vis absorption bands of the complexes in DMSO solutions—indication of the maxima λ in nm and the extinction coefficients (ε/M^−1^ cm^−1^). Shoulder (sh).

Compound	λ (nm) (ε/M^−1^ cm^−1^)
[Cu(HL)Cl]	268 (2.4 × 10^4^); 365 (2.2 × 10^4^); 456 (7.0 × 10^3^); 563 (2.0 × 10^3^)
[Cu(HL)_2_]	265 (5.2 × 10^4^); 365 (5.35 × 10^4^); 462 (1.1 × 10^4^); 567 (2.7 × 10^3^)
[Ni(HL)(acetate)]	283 (1.5 × 10^4^); 301 (1.4 × 10^4^); 379 (1.1 × 10^4^); 455 (1.2 × 10^4^); 516 (6.4 × 10^3^); 563 (3.6 × 10^3^)
[Ni(HL)_2_]	266 (3.8 × 10^4^); 366 (4.1 × 10^4^); 462 (1.75 × 10^4^); 512 (1.1 × 10^4^); 563 (5.0 × 10^3^)
[Ru(HL)Cl(DMSO)]	266 (2.1 × 10^4^); 370 (2.1 × 10^4^); 475 (4.5 × 10^3^); 770 (65)
[VO(HL)_2_]	267 (3.0 × 10^4^); 354 (sh, 3.0 × 10^4^); 376 (3.2 × 10^4^); 387 (sh, 2.85 × 10^4^); 540 (7.5 × 10^3^)
[Fe(HL)_2_Cl(H_2_O)]	270 (5.3 × 10^4^); 370 (5.0 × 10^4^); 387 (sh, 3.75 × 10^4^); 495 (1.2 × 10^4^); 665 (2.0 × 10^3^)

**Table 3:** Distribution coefficients (*D*_7.4_) measured for the studied compounds [pH = 7.4 (10 mM HEPES)].

	0 mM KCl	100 mM KCl
log*D*_7.4_ (ligand)	+2.8 ± 0.1	+3.0 ± 0.1
log*D*_7.4_ (Ru-complex)	+2.6 ± 0.1	+2.5 ± 0.1

**Table 4:** Proton dissociation constants (p*K*_a_) of the investigated compounds in 60% (*v*/*v*) DMSO/H_2_O mixture, in addition to the values predicted by Marvin software [55]. [*I* = 0.10 M (KCl)].

	Ligand (Predicted)	Ligand	2-Hydrazinobenzothiazole
p*K*_a1_	2.04	-	-
p*K*_a2_	2.90	1.99 ± 0.03	3.35 ± 0.03
p*K*_a3_	9.17	9.09 ± 0.04	10.35 ± 0.03
p*K*_a4_	10.09	12.99 ± 0.04	12.86 ± 0.03

**Table 5:** Half-inhibitory concentration (IC_50_) of [Ni(HL)(acetate)], [Ru(HL)Cl(DMSO)] and 5-FU towards human and murine colon cancer cell lines, after 48 h incubation.

	IC_50_ (µM) ± SD		
Cell Line	[Ni(HL)(Acetate)]	[Ru(HL)Cl(DMSO)]	5-FU
CT-26	11.0 ± 2.3	21.0 ± 4.2	2.7 ± 1.9
HCT-116	8.6 ± 2.0	16.6 ± 3.9	43.8 ± 2.3

## Data Availability

Data is available upon request.

## Supplementary Materials

The following supporting information can be downloaded at: https://www.mdpi.com/article/10.3390/pharmaceutics14122583/s1. Reference [88] is cited in the supplementary materials. Figure S1: (a) UV-Vis absorption spectrum of H_2_L (ca. 20 μM) in DMSO. (b) Fluorescence emission spectrum of H_2_L (ca. 20 μM) in DMSO (corrected by subtraction of the DMSO emission spectrum) (ε_ex_ = 475 nm). (c) H_2_L FTIR spectrum as KBr pellet. (d) UV-Vis absorption spectra of H_2_L in different solvents (DN is donor number). Figure S2: DFT optimised geometries of H_2_L conformers. Figure S3: FTIR spectra of [Cu(HL)Cl] (blue), [Ru(HL)Cl(DMSO)] (red) and [Fe(HL)_2_Cl(H_2_O)] (green) as CsI pellets. Figure S4: FTIR spectrum of H_2_L (blue), [Fe(HL)_2_Cl(H_2_O)] (red) and [VO(HL)_2_] (green) as KBr pellets. Figure S5: FTIR spectra of H_2_L (blue) and its Cu(II) complexes, [Cu(HL)Cl] (red) and [Cu(HL)_2_] (green), as KBr pellets. Figure S6: FTIR spectra of H_2_L (blue), [Ni(HL)(acetate)] (red) and [Ni(HL)_2_] (green) as KBr pellets. Figure S7: FTIR spectrum of H_2_L (blue) and [Ru(HL)Cl(DMSO)] (red) as KBr pellets. Figure S8: ^1^H NMR spectra of H_2_L (top blue line) and [Ni(HL)_2_] (down red line) in DMSO_d_6_ at 400 MHz. Figure S9: ^1^H NMR spectrum of [Ru(HL)Cl(DMSO)] in DMSO_d_6_ at a 400 MHz apparatus. Figure S10: First derivative EPR spectrum (experimental and simulated) of [Cu(HL)_2]_ (3 mM) in DMF at ca. 100 K. Figure S11: First derivative X- band EPR spectra measured and simulated for [V^IV^O(HL)_2_] (1 mM) in MeOH at ca. 100 K. Figure S12: UV-Vis spectra of H_2_L (blue, 34 µM), [Cu(HL)Cl] (red, 36 µM) and [Cu(HL)_2_] (green, 27 µM) in DMSO. Figure S13: UV-Vis absorption spectra of H_2_L (blue, 34 µM), [Ni(HL)(acetate)] (red, 30 µM) and [Ni(HL)_2_] (green, 28 µM) in DMSO. Figure S14: UV-Vis absorption spectra of H_2_L (blue, 34 μM), [Ru(HL)Cl(DMSO)] (red, 35 μM), [VO(HL)_2_] (green, 38 µM) and [Fe(HL)_2_Cl(H_2_O)] (purple, 31 µM) in DMSO. Inset: expansion of the [Ru(HL)Cl(DMSO)] spectrum in the 600–850 nm region showing a low intensity absorption band centred at 760 nm as predicted by DFT calculations. Figure S15: Studies of aqueous stability of the complexes in the absence (on the left) and in the presence of equimolar amount of BSA (on the right). From top to bottom: [Ni(HL)(acetate)] 15 µM and 24 µM; [Ni(HL)_2_] 9.5 µM and 22 µM; [Ru(HL)Cl(DMSO)] 35 µM and 22 µM; [VO(HL)_2_] 25 µM and 22.5 µM. Figure S16: Time-dependent UV-vis spectra of the ligand in DMSO, protected from light. [*c*_ligand_ = 122 μM; *ℓ* = 2 mm; *T* = 25.0 °C]. Inset: variation of A_474_ nm vs. time. Figure S17: Time-dependent UV-vis spectra of the ruthenium complex in DMSO. (A) Irradiated continuously with diffuse light. (B) Protected from light. [c_complex_ = 55 μM; ℓ = 5 mm; T = 25.0 °C]. Figure S18: Aromatic region of the time-dependent ^1^H NMR spectra from bottom to top: ligand protected from light, freshly dissolved in DMSO-d_6_; after 2.5 days; after 1 week; after 2.5 days irradiated; 2-hydrazinobenzothiazole. [c_ligand_ = c_2-hydrazinobenzothiazole_ = 2 mM; t = 25.0 °C]. Figure S19: UV-vis spectrum for the n-octanol phase. Charge transfer band of the Ru-complex before and after mixing and shaking with water phase. [c_complex_ = 100 μM; ℓ = 5 mm; t = 25.0 °C]. Figure S20: (A) pH-dependent UV-vis spectra of the ligand in the pH-range 1.65–12.41 in 30% (*v*/*v*) DMSO/water mixture. (B) Change of the baseline during the titration indicating precipitation. [c_ligand_ = 4.67 μM; I = 0.10 M (KCl); ℓ = 5 cm; t = 25.0 °C]. Figure S21: ^1^H NMR spectra of the Ru complex and the ligand in a) DMSO-d_6_ and b) in 60% (*v*/*v*) DMSO-d_6_/aqueous buffer mixture (pH = 7.4 (HEPES)). (The low quality of the spectra recorded in the latter solvent is connected to the limited solubility of the compounds that resulted in low concentration and precipitation in the NMR tubes). [c_complex_ = c_ligand_ = 2 mM; t = 25.0 °C]. Figure S22: Antiproliferative effect of a panel of metal-based complexes towards murine (B16F10) and human (A375) melanoma cell lines, human epidermal keratinocytes (HaCat) and murine (CT-26) and human (HCT-116) colon cancer cell lines. All compounds were tested at 20 and 40 µM. Cell viability was determined by MTT assay after a 48 h incubation period. Figure S23: Antiproliferative effect of the ligand H_2_L towards murine (CT-26) and human (HCT-116) colon cancer cell lines. Cell viability was determined by MTT assay after a 48 h incubation period. Table S1: Half-inhibitory concentration (IC_50_) of H_2_L towards human and murine colon cancer cell lines, after a 48 h incubation period.
