# Supplementary material for: Metal Coordination and Biological Screening of a Schiff Base Derived from 8-Hydroxyquinoline and Benzothiazole
Source: Pharmaceutics. 2022 Nov 24;14(12):2583. doi: 10.3390/pharmaceutics14122583 (PMC9785144; doi:10.3390/pharmaceutics14122583)
Supplement: Supplementary file 1 [file pharmaceutics-14-02583-s001.zip › pharmaceutics-2031390-supplementary.pdf]

Supplemental Material

Metal Coordination and Biological Screening of a Schiff Base Derived from 8-Hydroxyquinoline and Benzothiazole

Nádia Ribeiro [1,†], Pedro F. Farinha [2,†], Jacinta O. Pinho [2], Hugo Luiz [2], János P. Mészáros [3], Adelino M. Galvão [1], João Costa Pessoa [1], Éva A. Enyedy [3], Catarina Pinto Reis [2], Isabel Correia [1,*] and Maria Manuela Gaspar [2,*]

[1] Centro de Química Estrutural, Institute of Molecular Sciences and Departamento de Engenharia Química, Instituto Superior Técnico, Universidade de Lisboa, Av. Rovisco Pais, 1, 1049-001 Lisboa, Portugal

[2] Research Institute for Medicines, iMed.ULisboa, Faculty of Pharmacy, Universidade de Lisboa, 1649-003 Lisboa, Portugal

[3] MTA-SZTE Lendület Functional Metal Complexes Research Group, Department of Inorganic and Analytical Chemistry, University of Szeged, Dóm tér 7, H-6720 Szeged, Hungary

* Correspondence: icorreia@tecnico.ulisboa.pt (I.C.); mgaspar@ff.ulisboa.pt (M.M.G.)

† These authors contributed equally to this work.

Synthesis of H_2L

A mild oxidation of 8-hydroxy-2-methyl-quinoline with SeO_2, refluxing it overnight in 1,4-dioxane containing 2% (v/v) water, was used to obtain the 2-carbaldehyde-8-hydroxyquinoline, as reported in the literature [1]. An ethyl acetate-petroleum ether mixture was used to selectively precipitate the selenium impurities, affording a clear yellow solution of the desired carbaldehyde. After solvent removal, the aldehyde was obtained with enough purity to synthesise the ligand precursor.

In a reaction vessel, 1.25 mmol of 2-carbaldehyde-8-hydroxyquinoline was solubilized in ethanol (EtOH) and some drops of glacial acetic acid were added. Then, 1.25 mmol of 2-hydrazinobenzothiazole was added with constant stirring and the "coffee cream" colour mixture was left at room temperature (r.t.) under stirring for 6 h. A yellow solid formed, was collected by filtration and washed with ice-cold EtOH. The solid was dried under vacuum in a desiccator over silica-gel.

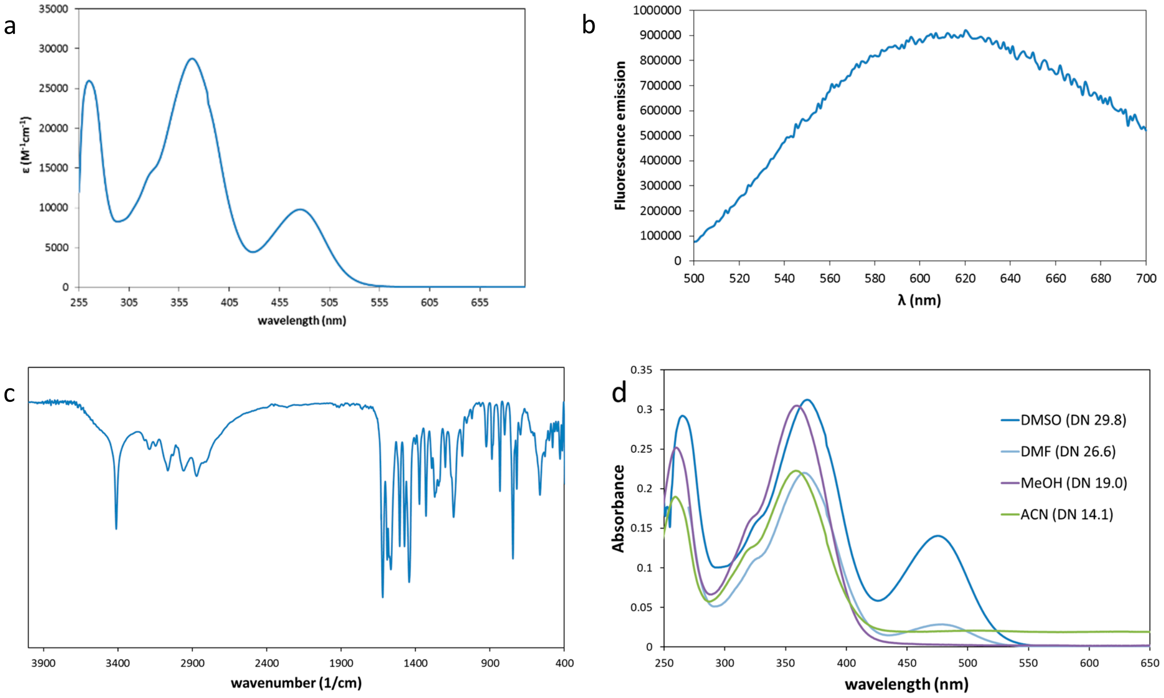

Figure S1. (a) UV-Vis absorption spectrum of H_2L (*ca.* 20 μM) in DMSO. (b) Fluorescence emission spectrum of H_2L (*ca.* 20 μM) in DMSO (corrected by subtraction of the DMSO emission spectrum) (λ_{ex} = 475 nm). (c) H_2L FTIR spectrum as KBr pellet. (d) UV-Vis absorption spectra of H_2L in different solvents (DN is donor number).

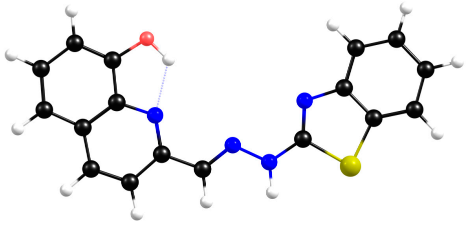

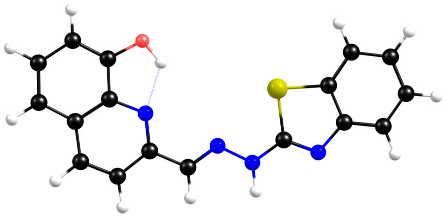

A **B**

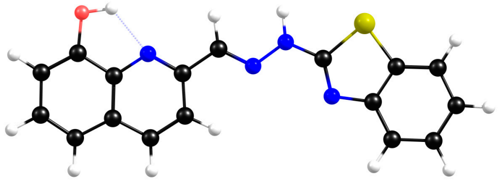

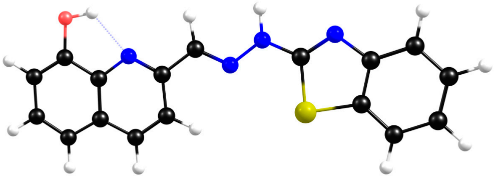

C **D**

Figure S2. DFT optimized geometries of H_2L conformers.

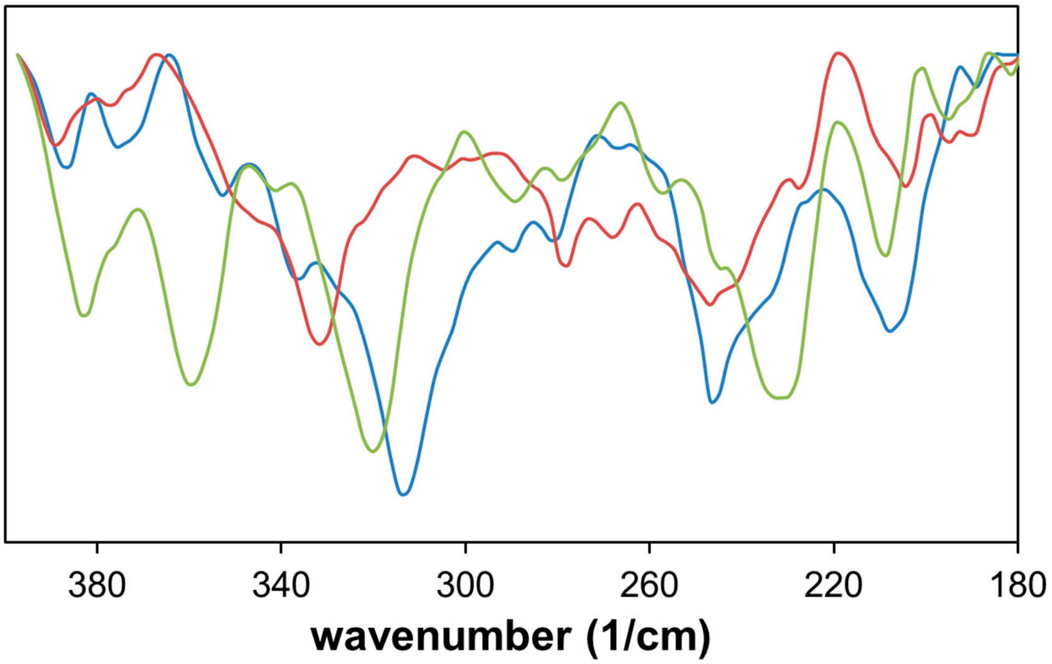

Figure S3. FTIR spectra of [Cu(HL)Cl] (blue), [Ru(HL)Cl(DMSO)] (red) and [$Fe(HL)_2Cl(H_2O)$] (green) as CsI pellets.

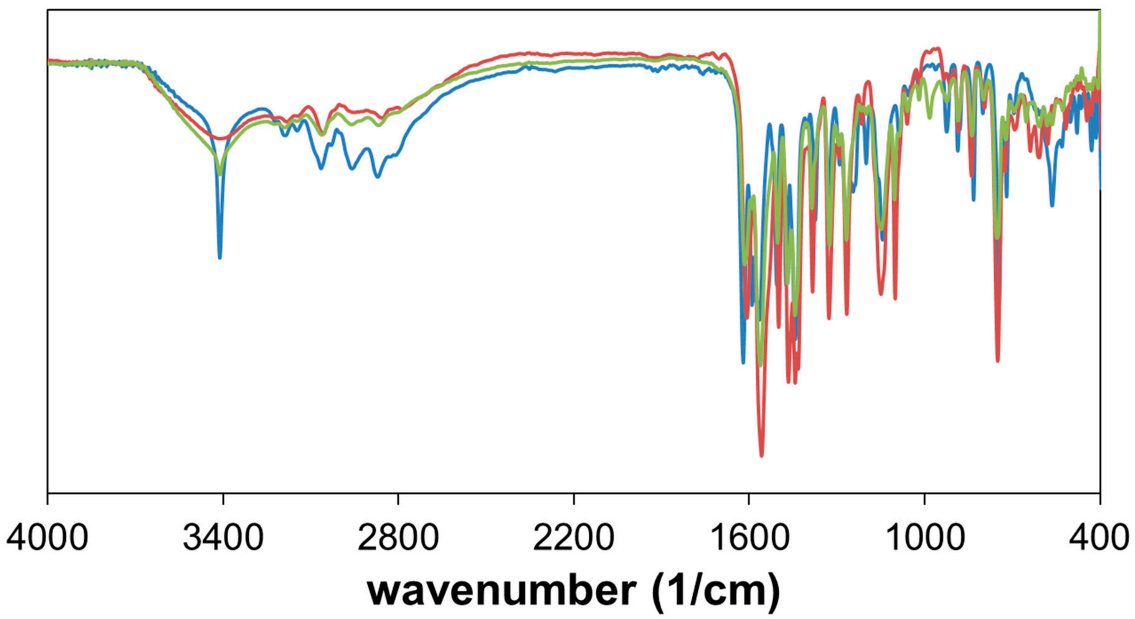

Figure S4. FTIR spectrum of H_2L (blue), $[Fe(HL)_2Cl(H_2O)]$ (red) and $[VO(HL)_2]$ (green) as KBr pellets.

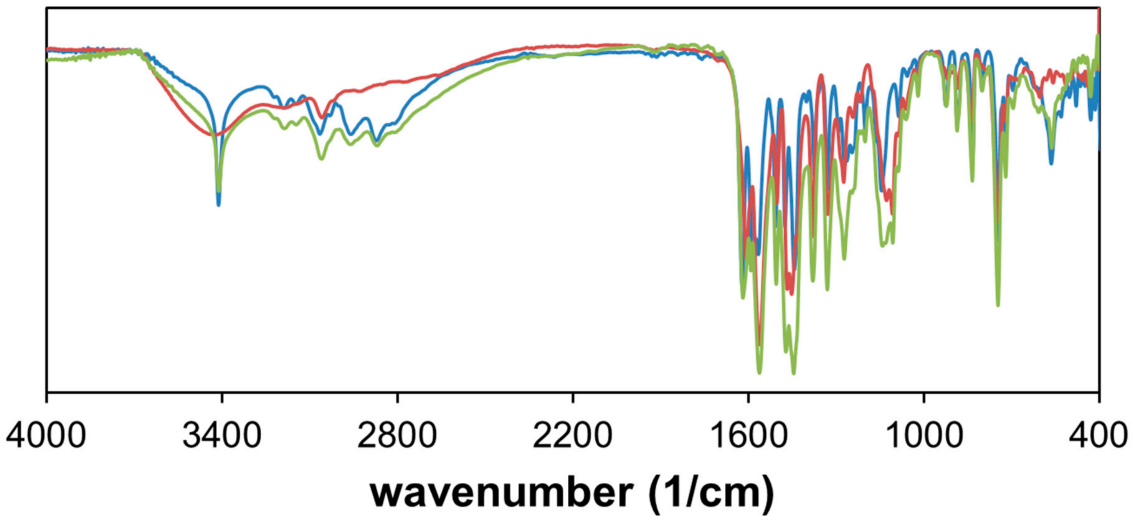

Figure S5. FTIR spectra of H_2L (blue) and its Cu(II) complexes, $[Cu(HL)Cl]$ (red) and $[Cu(HL)_2]$ (green), as KBr pellets.

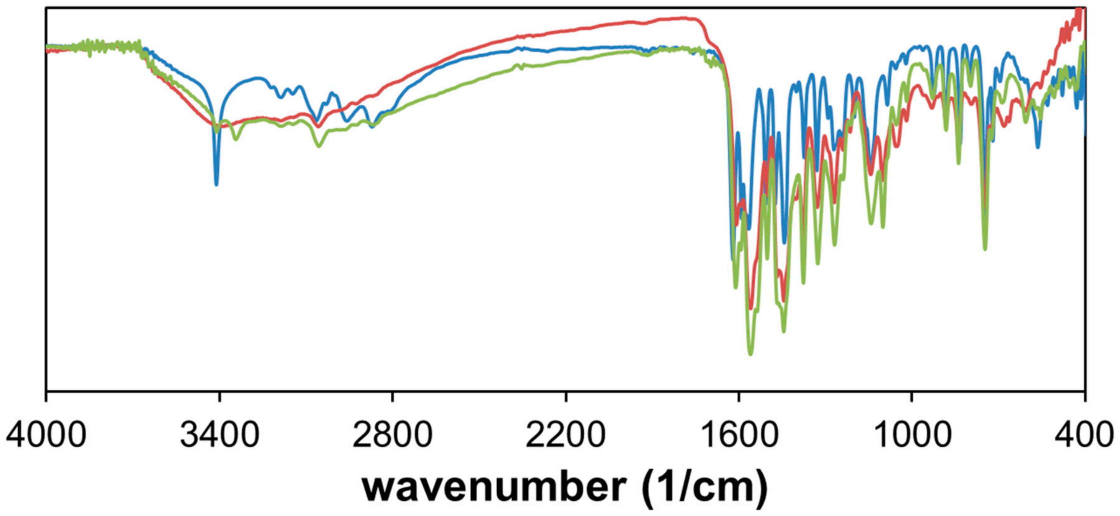

Figure S6. FTIR spectra of H_2L (blue), [Ni(HL)(acetate)] (red) and $[Ni(HL)_2]$ (green) as KBr pellets.

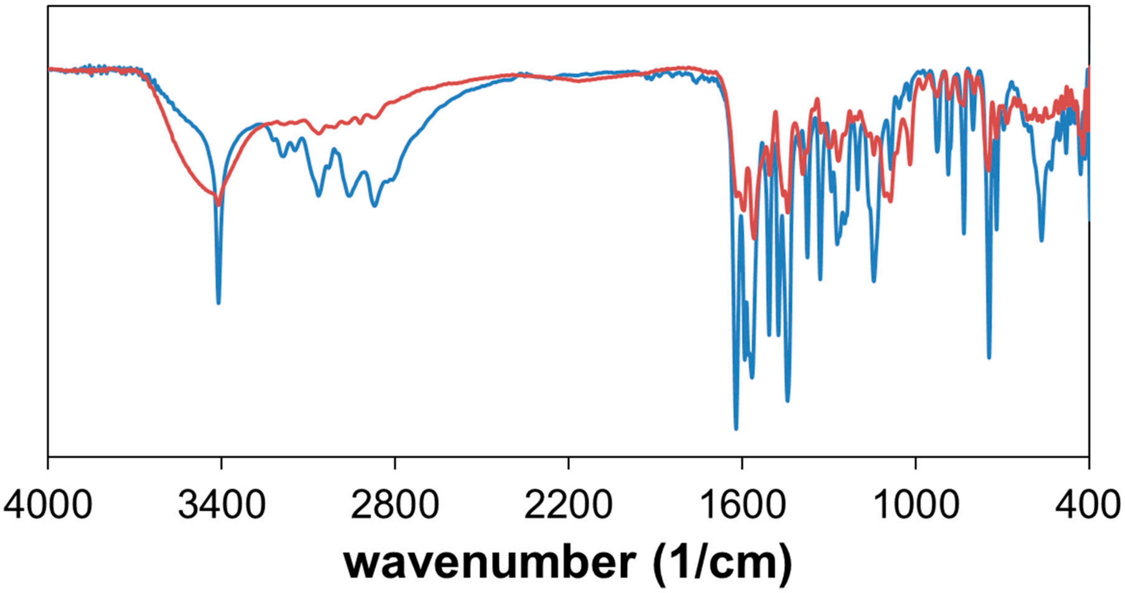

Figure S7. FTIR spectrum of H_2L (blue) and [Ru(HL)Cl(DMSO)] (red) as KBr pellets.

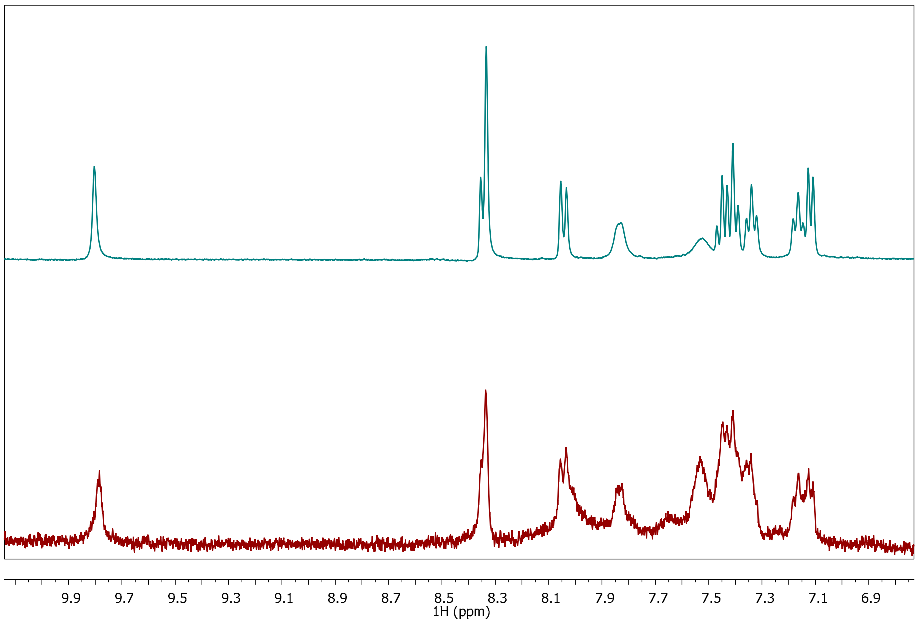

Figure S8. ^{1}H NMR spectra of H_2L (top blue line) and $[Ni(HL)_2]$ (down red line) in DMSO_d_6 at 400 MHz.

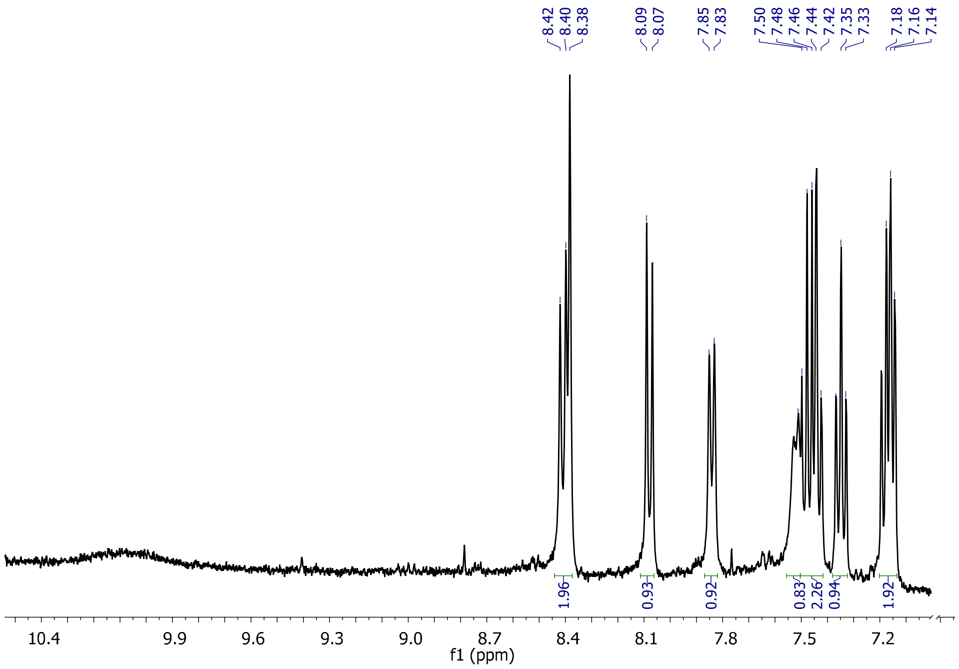

Figure S9. ^{1}H NMR spectrum of [Ru(HL)Cl(DMSO)] in DMSO_d_6 at a 400 MHz apparatus.

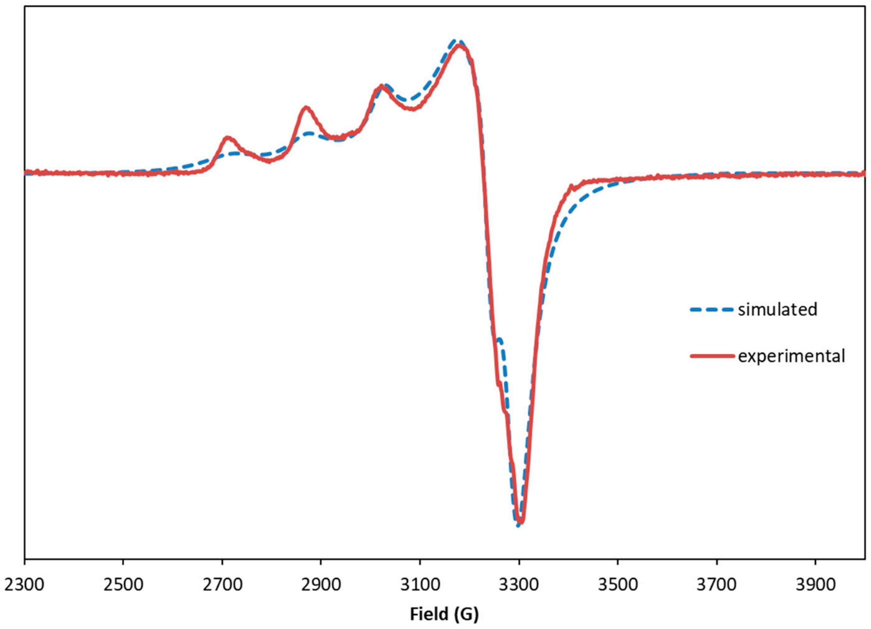

Figure S10. First derivative EPR spectrum (experimental and simulated) of $[Cu(HL)_2]$ (3 mM) in DMF at *ca.* 100 K.

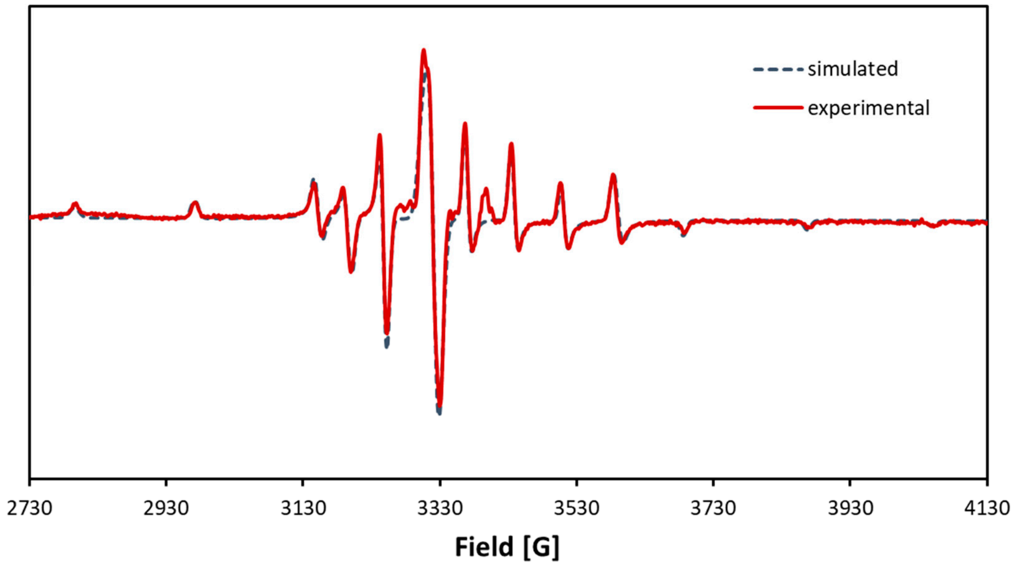

Figure S11. First derivative X- band EPR spectra measured and simulated for $[V^{IV}O(HL)_2]$ (1 mM) in MeOH at *ca.* 100 K.

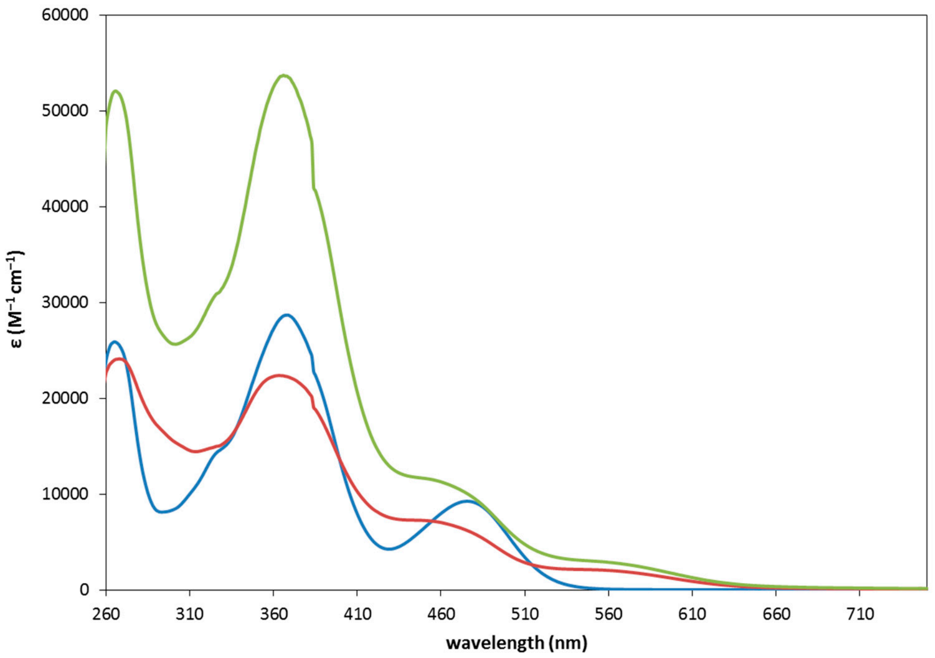

Figure S12. UV-Vis spectra of H_2L (blue, 34 μM), [Cu(HL)Cl] (red, 36 μM) and $[Cu(HL)_2]$ (green, 27 μM) in DMSO.

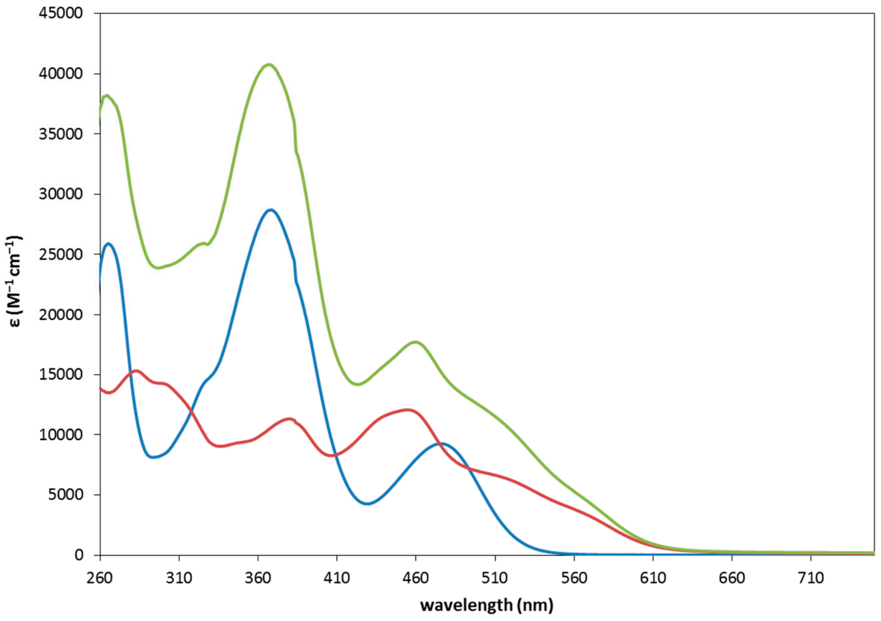

Figure S13. UV-Vis absorption spectra of H_2L (blue, 34 μM), [Ni(HL)(acetate)] (red, 30 μM) and $[Ni(HL)_2]$ (green, 28 μM) in DMSO.

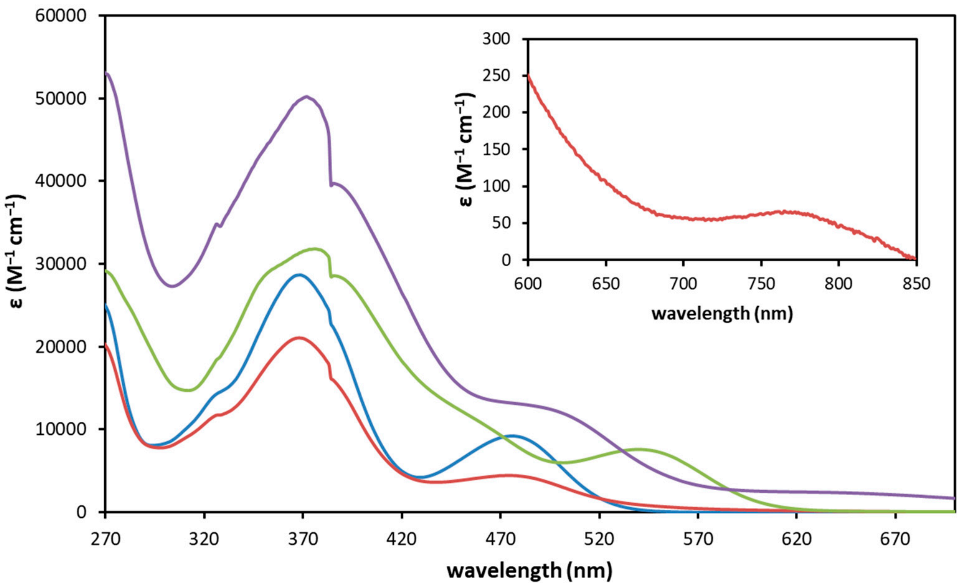

Figure S14. UV-Vis absorption spectra of H_2L (blue, 34 μM), [Ru(HL)Cl(DMSO)] (red, 35 μM), [VO(HL)$_2$] (green, 38 μM) and [Fe(HL)$_2$Cl(H_2O)] (purple, 31 μM) in DMSO. Inset: expansion of the [Ru(HL)Cl(DMSO)] spectrum in the 600 – 850 nm region showing a low intensity absorption band centered at 760 nm as predicted by DFT calculations.

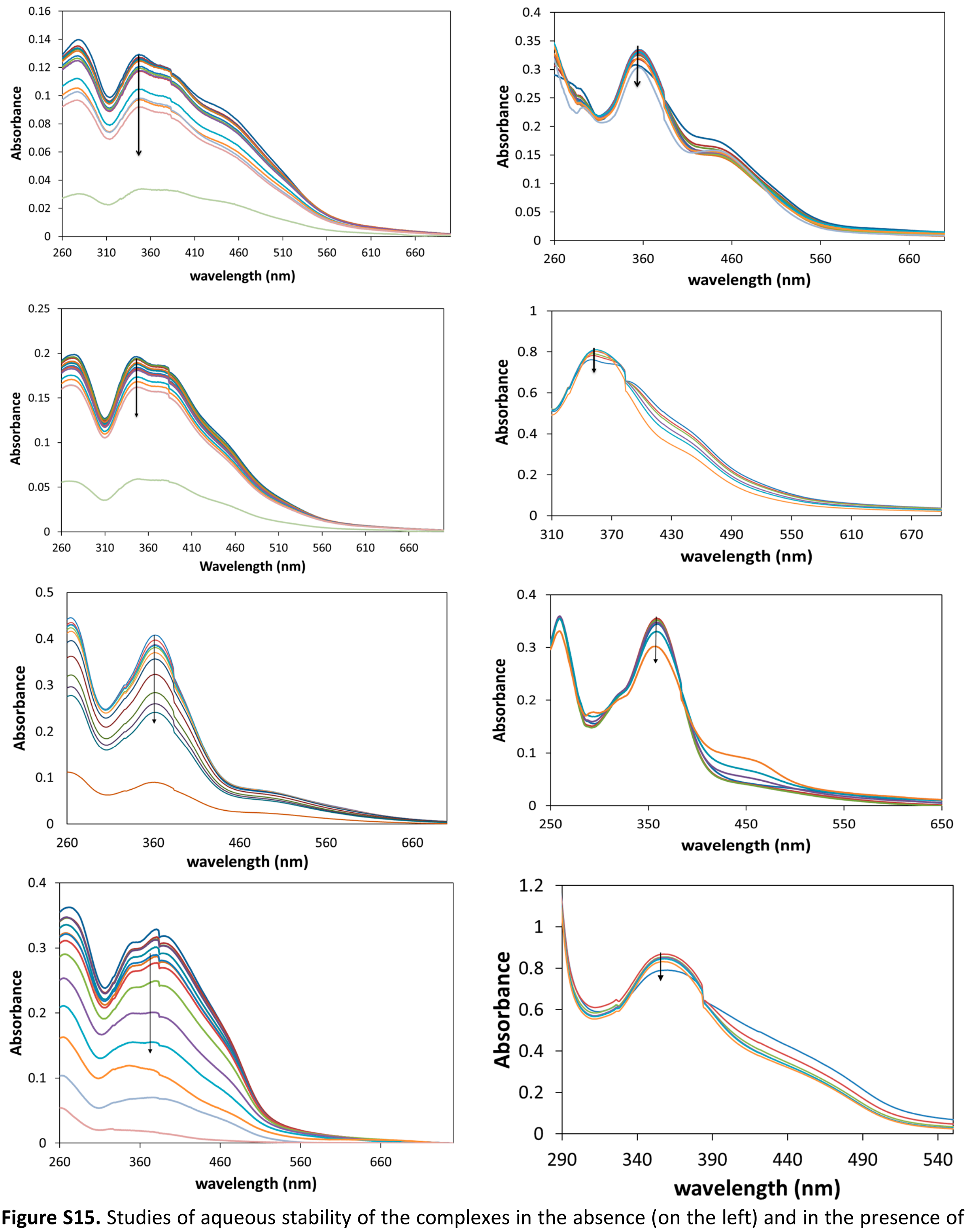

Figure S15. Studies of aqueous stability of the complexes in the absence (on the left) and in the presence of equimolar amount of BSA (on the right). From top to bottom: [Ni(HL)(acetate)] 15 µM and 24 µM; $[Ni(HL)_2]$ 9.5 µM and 22 µM; [Ru(HL)Cl(DMSO)] 35 µM and 22 µM; $[VO(HL)_2]$ 25 µM and 22.5 µM.

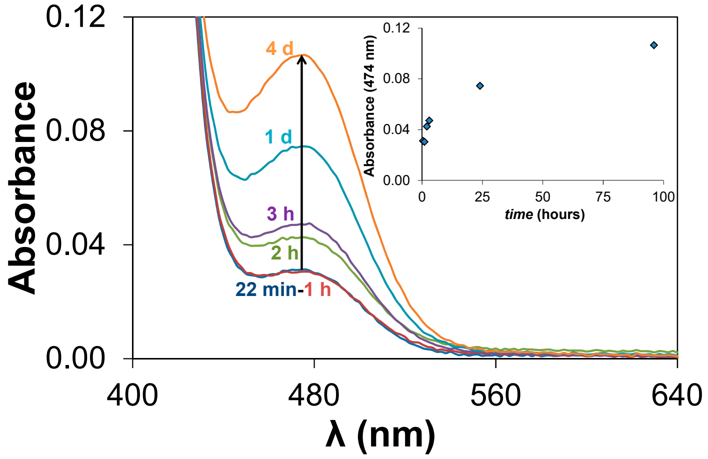

Figure S16. Time-dependent UV-vis spectra of the ligand in DMSO, protected from light. [c_{ligand} = 122 µM; ℓ = 2 mm; T = 25.0°C]. Inset: variation of $A_{474\text{ nm}}$ vs. time.

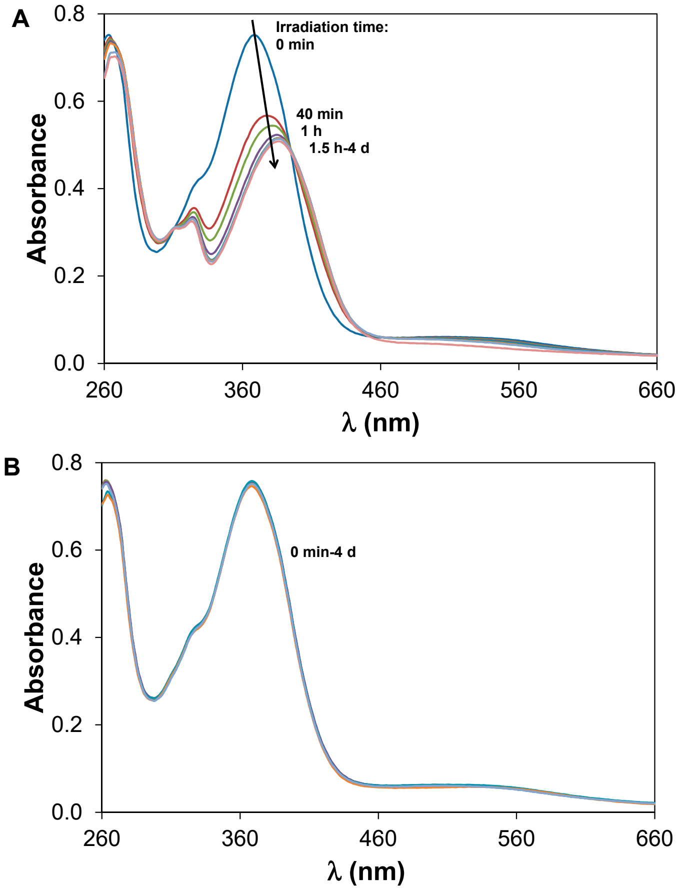

Figure S17. Time-dependent UV-vis spectra of the ruthenium complex in DMSO. A) Irradiated continuously with diffuse light. B) Protected from light. [c_{complex} = 55 µM; ℓ = 5 mm; t = 25.0°C].

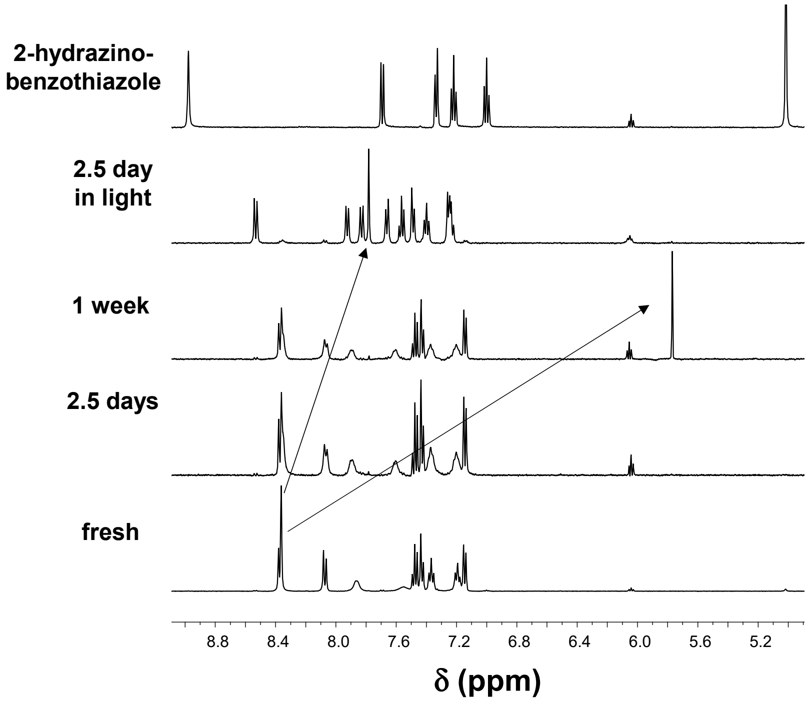

Figure S18. Aromatic region of the time-dependent ^{1}H NMR spectra from bottom to top: ligand protected from light, freshly dissolved in DMSO-d_6; after 2.5 days; after 1 week; after 2.5 days irradiated; 2-hydrazinobenzothiazole. [c_{ligand} = $c_{\text{2-hydrazinobenzothiazole}}$ = 2 mM; t = 25.0°C].

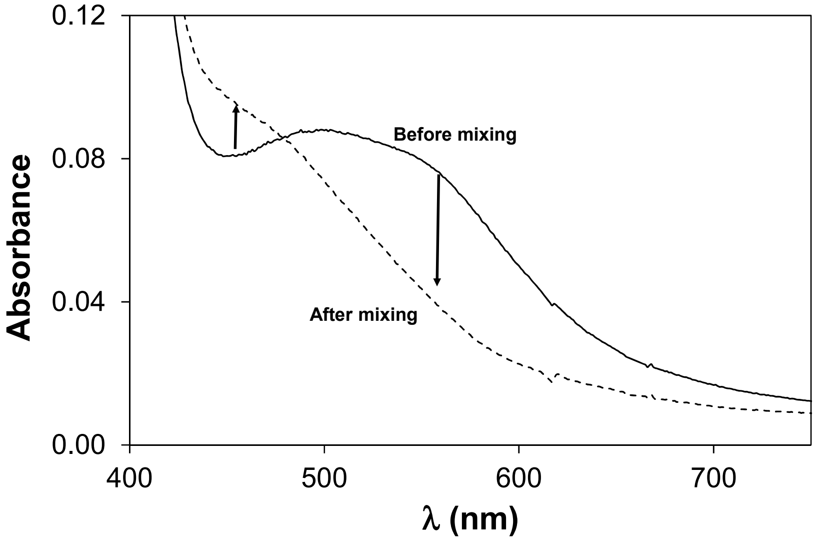

Figure S19. UV-vis spectrum for the *n*-octanol phase. Charge transfer band of the Ru-complex before and after mixing and shaking with water phase. [c_{complex} = 100 µM; ℓ = 5 mm; t = 25.0°C].

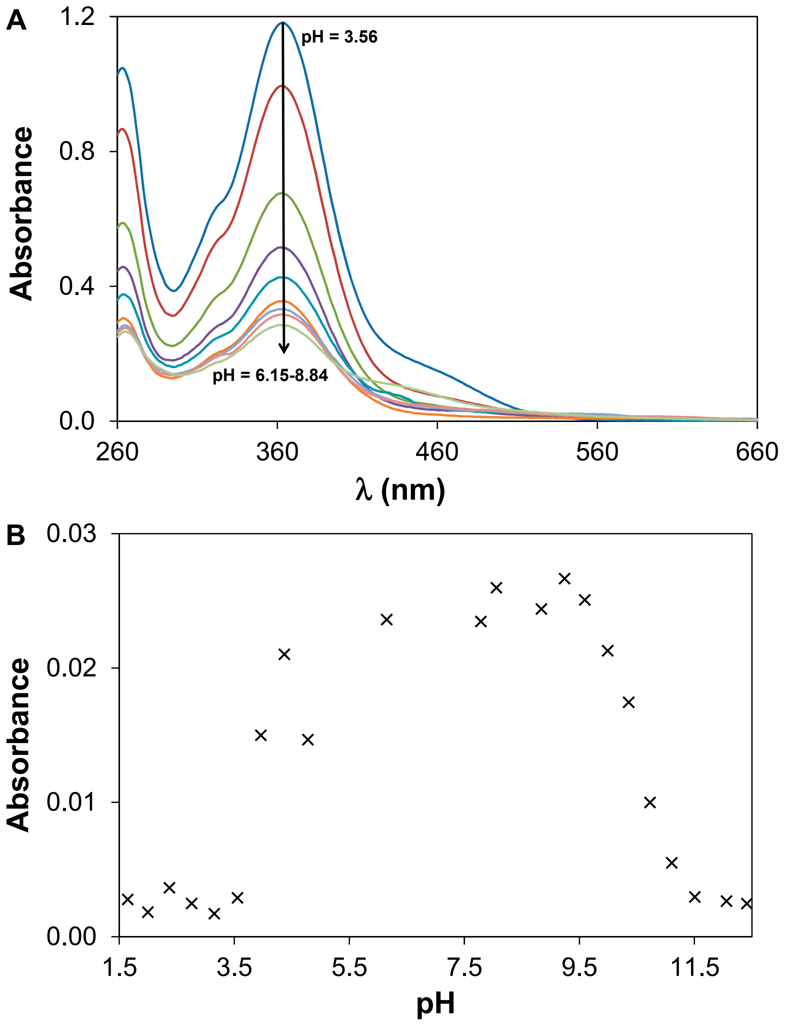

Figure S20. A) pH-dependent UV-vis spectra of the ligand in the pH-range 1.65–12.41 in 30% (v/v) DMSO/water mixture. B) Change of the baseline during the titration indicating precipitation. [c_{ligand} = 4.67 µM; I = 0.10 M (KCl); ℓ = 5 cm; t = 25.0°C].

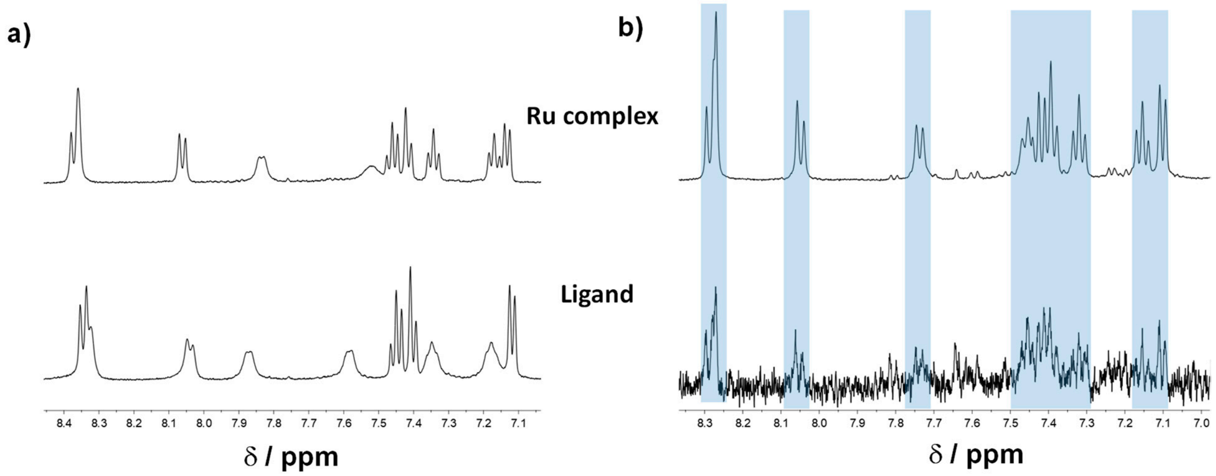

Figure S21. ^{1}H NMR spectra of the Ru complex and the ligand in a) DMSO-d_6 and b) in 60% (v/v) DMSO-d_6/aqueous buffer mixture (pH = 7.4 (HEPES)). (The low quality of the spectra recorded in the latter solvent is connected to the limited solubility of the compounds that resulted in low concentration and precipitation in the NMR tubes). [$c_{complex}$ = c_{ligand} = 2 mM; t = 25.0°C].

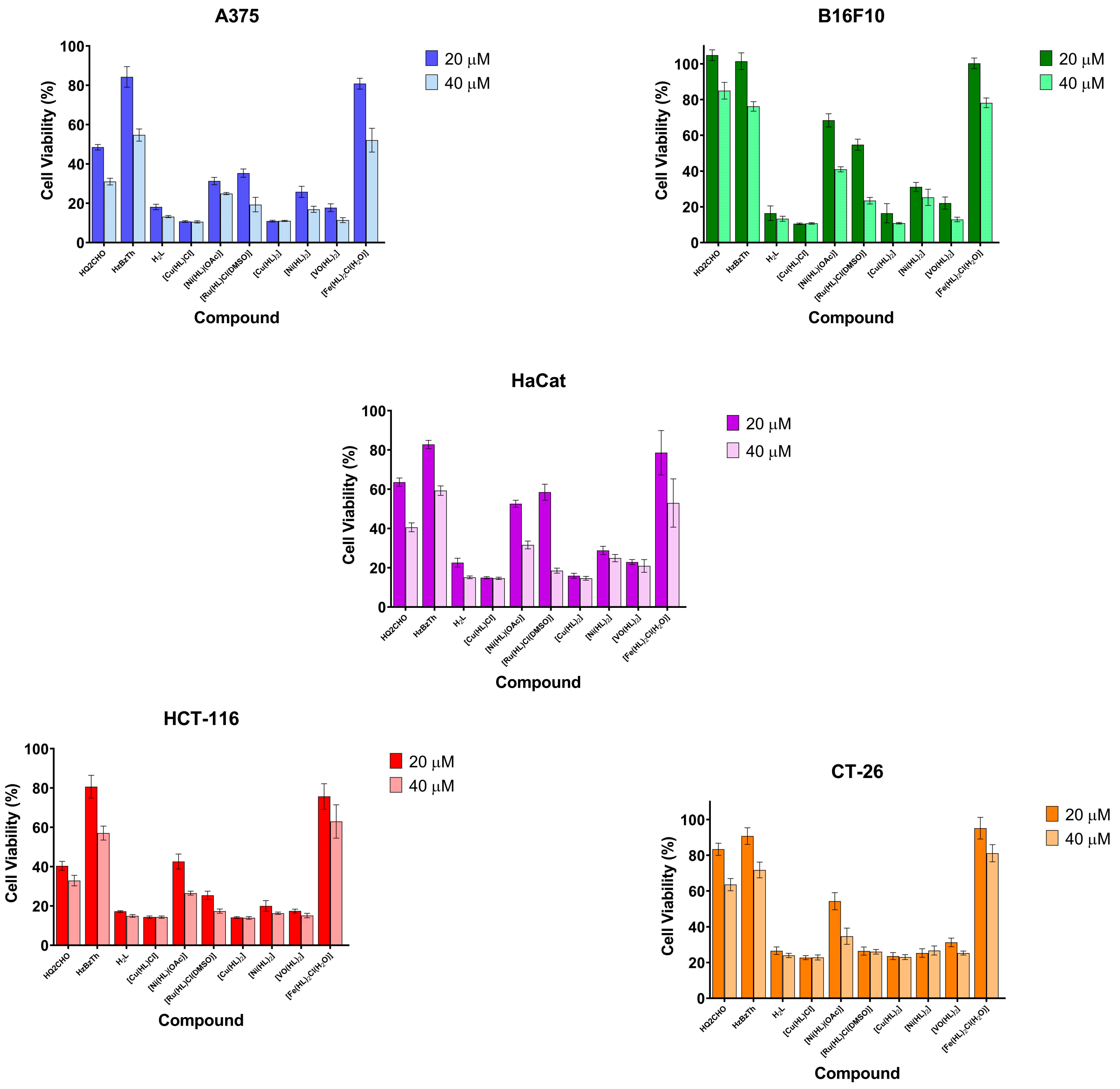

Figure S22. Antiproliferative effect of a panel of metal-based complexes towards murine (B16F10) and human (A375) melanoma cell lines, human epidermal keratinocytes (HaCat) and murine (CT-26) and human (HCT-116) colon cancer cell lines. All compounds were tested at 20 and 40 μM. Cell viability was determined by MTT assay after a 48 h incubation period.

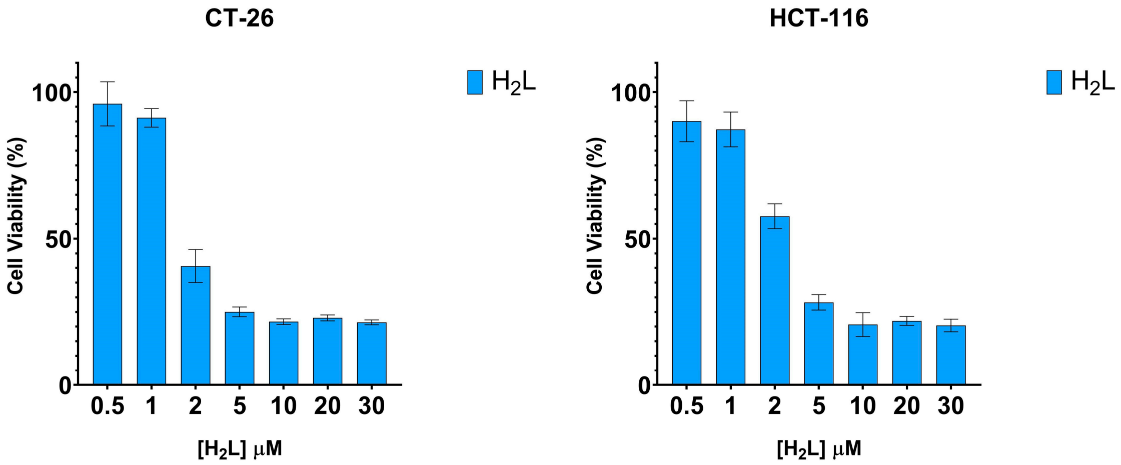

Figure S23. Antiproliferative effect of the ligand H_2L towards murine (CT-26) and human (HCT-116) colon cancer cell lines. Cell viability was determined by MTT assay after a 48 h incubation period.

Table S1. Half-inhibitory concentration (IC_{50}) of H_2L towards human and murine colon cancer cell lines, after a 48 h incubation period.

	IC_{50} (µM)	
**Compound**	**CT-26**	**HCT-116**
H_2L	1.8 ± 0.4	3.0 ± 0.8

References

[1] - M. Hassani, W. Cai, D.C. Holley, J.P. Lineswala, B.R. Maharjan, G.R. Ebrahimian, H. Seradj, M.G. Stocksdale, F. Mohammadi, C.C. Marvin, J.M. Gerdes, H.D. Beall, M. Behforouz, Novel Lavendamycin Analogues as Antitumor Agents: Synthesis, in Vitro Cytotoxicity, Structure−Metabolism, and Computational Molecular Modeling Studies with NAD(P)H:Quinone Oxidoreductase 1, *J. Med. Chem.*, 48 (2005) 7733-7749.
